# Low Serum Lysophospholipids Predict Increased In‐Hospital Mortality in Patients With Acute Heart Failure

**DOI:** 10.1161/JAHA.125.043828

**Published:** 2026-01-19

**Authors:** Iva Klobučar, Gudrun Pregartner, Gerald Rechberger, Thomas O. Eichmann, Ines Potočnjak, Matias Trbušić, Hansjörg Habisch, Bastian Pfeifer, Tobias Madl, Andrea Berghold, Saša Frank, Vesna Degoricija

**Affiliations:** ^1^ Department of Cardiology Sisters of Charity University Hospital Center Zagreb Croatia; ^2^ Institute for Medical Informatics, Statistics und Documentation Medical University of Graz Graz Austria; ^3^ Institute of Molecular Biosciences University of Graz Graz Austria; ^4^ BioTechMed‐Graz Graz Austria; ^5^ Field of Excellence BioHealth University of Graz Graz Austria; ^6^ Core Facility Mass Spectrometry, Center for Medical Research Medical University of Graz Graz Austria; ^7^ Sisters of Charity University Hospital Center Zagreb Croatia; ^8^ School of Medicine Catholic University of Croatia Zagreb Croatia; ^9^ University of Zagreb School of Medicine Zagreb Croatia; ^10^ Medicinal Chemistry, Otto Loewi Research Center Medical University of Graz Graz Austria; ^11^ Gottfried Schatz Research Center, Molecular Biology and Biochemistry Medical University of Graz Graz Austria

**Keywords:** acute heart failure, biomarkers, lipidomics, mass spectrometry, outcome, Heart Failure, Lipids and Cholesterol, Biomarkers

## Abstract

**Background:**

New biomarkers are needed to improve risk prediction in patients with acute heart failure. We aimed to identify serum lipids with prognostic value and clinical utility in patients hospitalized due to acute heart failure.

**Methods:**

Targeted mass spectrometry–based lipidomics was performed on serum samples from 315 (discovery) and 139 (validation) patients prospectively enrolled in 2 observational acute heart failure studies. Prognostic lipids were identified by consolidating orthogonal partial least squares discriminant analysis, least absolute shrinkage and selection operator regression, and random forest (Boruta) results into a single ranking using TopKSignal.

**Results:**

In the discovery cohort, lysophosphatidylethanolamine 20:4, lysophosphatidylcholine 20:4, lysophosphatidylcholine 14:0, and lysophosphatidylethanolamine 18:1 were most strongly associated with in‐hospital mortality. Serum concentrations were significantly lower in nonsurvivors, and these inverse associations remained significant after adjustment in both cohorts, except for lysophosphatidylethanolamine 18:1 after adjustment for OPTIMIZE‐HF (Organized Program to Initiate Lifesaving Treatment in Hospitalized Patients With Heart Failure) in the validation cohort. These lipids demonstrated moderate discriminative performance (area under the curve, 0.72–0.76 in the discovery and 0.71–0.77 in the validation cohort) and provided additional prognostic value beyond ADHERE (Acute Decompensated Heart Failure National Registry), GWTG‐HF (Get With The Guidelines Heart Failure), and OPTIMIZE‐HF (Δarea under the curve, 0.04–0.17) scores. Decision curve analyses showed improved net clinical benefit for mortality prediction across threshold probabilities of 12% to 35% in the discovery and 15% to 28% in the validation cohort.

**Conclusions:**

Low serum levels of lysophosphatidylethanolamine 20:4, lysophosphatidylcholine 20:4, lysophosphatidylcholine 14:0, and lysophosphatidylethanolamine 18:1 were independently associated with in‐hospital mortality in acute heart failure. These findings suggest that specific lysophospholipids may serve as novel prognostic biomarkers, warranting validation in larger studies to confirm their clinical applicability.

## Graphical abstract

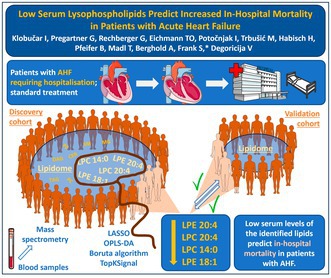

**Nonstandard Abbreviations and Acronyms**
AHF: acute heart failure

**Clinical Perspective**

**What is New?**

- Lysophosphatidylethanolamine 20:4, lysophosphatidylcholine 20:4, lysophosphatidylcholine 14:0, and lysophosphatidylethanolamine 18:1 are potential new biomarkers predictive of in‐hospital mortality in patients with acute heart failure.
- These lipids improved discrimination for in‐hospital mortality when considered on top of the established clinical scores ADHERE (Acute Decompensated Heart Failure National Registry), GWTG‐HF (Get With The Guidelines Heart Failure), or OPTIMIZE‐HF (Organized Program to Initiate Lifesaving Treatment in Hospitalized Patients With Heart Failure).

**What Are the Clinical Implications?**

- The identified lipids could complement existing biomarkers to improve risk estimation in patients with acute heart failure.

Heart failure (HF) is a complex clinical syndrome characterized by elevated intracardiac pressures and reduced cardiac capacity to deliver oxygen and nutrients to peripheral tissues. Acute HF (AHF) denotes the rapid onset or worsening of HF signs and symptoms.^1^, ^2^, ^3^ Despite advances in prognostic models and therapeutic strategies, risk prediction in AHF remains suboptimal, and mortality rates are high.^4^ Therefore, identifying and implementing novel biomarkers with strong prognostic value are crucial to improve risk stratification and clinical outcomes in AHF.

Circulating lipoproteins, extracellular vesicles, and serum proteins serve as major carriers of serum lipids.^5^ Serum lipid levels reflect the dynamic balance among lipid production, use, and degradation, processes regulated by intra‐ and extracellular enzymes, intestinal absorption, and the biosynthetic activity of enterocytes and hepatocytes.^5^, ^6^

In HF, hemodynamic impairment with accompanying tissue hypoperfusion and congestion, elevated natriuretic peptides and catecholamines, chronic low‐grade inflammation, insulin resistance, impaired nutrient absorption, reduced appetite, and decreased hepatic and intestinal biosynthetic activity contribute to metabolic dysfunction and catabolic dominance, core features of HF pathophysiology.^7^ Given the close interrelation between hemodynamic and metabolic disturbances in HF^8^ and the multifactorial regulation of lipid bioavailability, serum lipid profiles may mirror the degree of metabolic and hemodynamic impairment and thus reflect disease severity.

Previous studies have investigated lipidomic signatures and the diagnostic and prognostic relevance of lipids in chronic HF.^9^, ^10^, ^11^, ^12^, ^13^, ^14^, ^15^, ^16^, ^17^, ^18^, ^19^, ^20^ However, only 2 have examined associations between serum lipids and long‐term outcomes in AHF,^21^, ^22^ and none have evaluated their relationship with in‐hospital outcomes. To address this gap, we applied mass spectrometry (MS)–based serum lipidomics to identify lipids that may predict in‐hospital mortality in patients with AHF.

## METHODS

### Data Availability Statement

The data that support the findings of this study are available from the corresponding author upon reasonable request.

### Study Cohorts

Patients with signs and symptoms of AHF requiring hospitalization, from both discovery and validation cohort, were enrolled prospectively and consecutively in observational studies, as they presented to the Emergency Department of the Sisters of Charity University Hospital Center in Zagreb, Croatia.

Patients in the discovery cohort (N=315) were recruited between March 2018 and February 2021^23^ and in the validation cohort (N=152) between November 2013 and February 2015.^24^ The applied diagnostic criteria and treatment protocols for AHF for both cohorts followed the European Society of Cardiology guidelines that were valid at the time of participants’ enrollment.^1^, ^25^ Patient history data were recorded, physical examination done, and venous blood obtained at the time the patients presented to the emergency department, before the application of any medication. Echocardiography examination was performed within the first day of hospitalization. The study protocols are described in detail in the previous reports.^23^, ^24^ From the validation cohort, 139 blood samples were available for the present lipidomics analyses. In‐hospital mortality was the primary outcome.

Eligible patients gave signed informed consent for participation. The study was approved by the local ethics committees of the Sisters of Charity University Hospital Center, Zagreb, Croatia (EP 2258/18‐10 for discovery, EP 15389/13‐4 for validation), and the Medical University of Graz, Graz, Austria (EK 33‐258 ex 20/21 for discovery, EK 29‐266 ex 16/17 for validation). The investigation conforms with the principles outlined in the Good Clinical Practice guidelines and the Declaration of Helsinki.^26^

### Preparation and Handling of Serum

Serum was obtained from blood samples after incubation at room temperature for 30 minutes, followed by centrifugation at 1800*g* for 10 minutes at 4 °C. The serum was divided into 1 mL aliquots and stored at −80 °C. For different analyses, aliquots were thawed on ice, transferred into prechilled tubes in volumes appropriate for the respective analytical methods, including MS, and refrozen at −80 °C until further use.

### Lipid Extraction and Profiling by MS

Lipids were extracted according to the method of Matyash et al.^27^ In brief, internal standard stock solution was added to a mixture of methyl‐tert‐butylether/methanol (10/3, vol/vol) (2 μL IST/700 μL methyl‐tert‐butylether/methanol). 700 μL of the methyl‐tert‐butylether/methanol solution were added to the sample. After vigorous shaking at 30 Hz for 10 minutes on a mixer mill (Retsch, Haan, Germany) at 4 °C, 400 μL of water was added and the samples were shaken on a thermomix (Eppendorf, Hamburg, Germany) at 4 °C for 15 minutes. After centrifugation (18 000*g*), 350 μL of the organic upper phase were collected and dried under a stream of nitrogen. Samples were dissolved in 100 μL of isopropanol/methanol/water (6/3/1, v/v/v) and chromatographically separated with a 1260 Infinity II UHPLC (Agilent, Waldbronn, Germany). Next, 2 μL were injected and separated on a BEH C18 Column (150×2.1, 1.7 μm) (Waters, Manchester, UK) that was kept at 50 °C. A binary gradient starting at 50% solvent A (water, phosphoric acid [8 μM], ammonium acetate [10 mM] and formic acid [0.1 vol%]) and reaching 100% solvent B (isopropanol, phosphoric acid [8 μM], ammonium acetate [10 mM] and formic acid [0.1 vol%]) within 22 minutes at a total run time of 30 minutes and a flow rate of 150 μL/min was used. MS analysis was performed in positive electrospray ionization mode (3.5 kV, 300 °C gas temperature) using dynamic multiple reaction monitoring mode with a cycle time of 700 ms. Further details including the multiple reaction monitoring list can be found in the supplement (Supplemental Methods).

### Statistical Analysis

We considered all lipids for analysis that were measured in both the discovery and the validation cohort (N=98 lipids) and that had at least 80% of data above the detection limit (only 1 further lipid excluded). Lipid data were log2‐transformed and values under the detection limit were imputed using the QRILC algorithm, a missing data imputation method that performs the imputation of left‐censored missing data using random draws from a truncated distribution with parameters estimated using quantile regression, that is implemented in the R package imputeLCMD.

We then performed 3 methods of variable selection, namely (1) orthogonal partial least squares discrimination analyses, (2) least absolute shrinkage and selection operator regression, and (3) a random forest approach using the Boruta algorithm. For (1), the analyses, together with the associated data consistency checks and 7‐fold cross‐validation were performed using MetaboAnalyst version 5.0.^28^ For (2), we performed least absolute shrinkage and selection operator regression, a penalized regression method that is capable of variable selection, using the function glmnet() from the homonymous R package. We did 500 replications and for each lipid noted how often it was selected in the process. For (3), we performed 10 runs of the Boruta algorithm for feature selection, which by default uses random forest classification to perform an iterative top‐down search for relevant features by comparing original attribute importance with the importance of so‐called shadow attributes, which are created by permutation of the original ones. In each run the algorithm gives a final decision of “confirmed,” “tentative,” or “rejected” for each feature. Finally, we consolidated the results from the three selection methods using the R‐package TopKSignal^29^ to obtain one final ranking of lipids. TopKSignal computes the consensus rank using a signal‐plus‐noise model for signal reconstruction, which aims to recover the true underlying ranking from a set of noisy rankings. In contrast, standard rank aggregation methods typically do not account for the specific noise characteristics in the ranking data, making them more susceptible to errors or inconsistencies. Additionally, the approach provides measures of (un)certainty for the estimated consensus signals, which are valuable for assessing the reliability of results from feature selection processes. We have created 10 replicates of the orthogonal partial least squares discrimination analysis and least absolute shrinkage and selection operator regression derived feature ranks while resolving ties randomly. The resulting 30 feature rankings (10 per method, including the 10 original Boruta rankings) were then used as input for TopKSignal.

The identified relevant lipids as well as other clinical and laboratory data are descriptively summarized as median and interquartile range (Q1, Q3) for continuous parameters, because most of these parameters had skewed distributions according to QQ plots, or absolute and relative frequencies for categorical parameters. Group differences were assessed with the Brunner–Munzel test or Fisher’s exact test. For the selected lipids, we performed receiver operating characteristics and logistic regression analysis to assess their prognostic values for in‐hospital mortality. For receiver operating characteristics analyses, we assessed the area under the curve (AUC) for each individual lipid, and for the individual lipids in combination with established scores the ADHERE (Acute Decompensated Heart Failure National Registry) risk tree,^30^ the American Heart Association GWTG‐HF (Get With the Guidelines‐Heart Failure) risk score),^31^ and the OPTIMIZE‐HF (Organized Program to Initiate Lifesaving Treatment in Hospitalized Patients With Heart Failure) risk‐prediction algorithm.^32^ We assessed improvement via change in AUC and decision curve analysis. We compared AUC and the net benefit of models incorporating the individual lipids on top of the clinical scores with models containing only the clinical scores. The Spearman correlation coefficient was used to assess correlations between the selected lipids and various clinical and laboratory parameters. *P* values <0.05 are considered significant. All analyses were conducted using R version 4.4.0. In particular, the packages pROC and Hmisc were used.

## RESULTS

### Study Cohorts

Age, sex, body mass index, and vital signs at hospital admission did not differ significantly between the discovery and validation cohort (Table 1). The majority of patients in both cohorts presented with New York Heart Association class III or IV functional status. Serum NT‐proBNP (N‐terminal pro‐brain natriuretic peptide) levels were comparable between cohorts but were significantly higher among non‐urvivors within each cohort. In the discovery cohort, a higher proportion of patients developed AHF on top of chronic HF. Compared with the validation cohort, patients in the discovery cohort exhibited slightly but significantly lower left ventricular ejection fraction and higher pulmonary systolic pressure, accompanied by more frequent lung rales and liver enlargement. They also had significantly lower serum albumin and lipid levels (except for high‐density lipoprotein cholesterol, which was higher) and mildly, but significantly, higher inflammatory markers, despite the absence of acute infection. Length of hospitalization (median 8.0 [interquartile range, 6.0–12.5] days in discovery versus 9.0 [interquartile range, 7.0–13.0] days in validation, *P*=0.070) and in‐hospital mortality (34 [10.8%] in discovery versus 21 [15.1%] in validation, *P*=0.213) were also similar between the cohorts.

**Table 1:** Comparison of Clinical and Laboratory Baseline Characteristics of Patients in the Discovery and Validation Cohort

Variable	Discovery cohort				Validation cohort				Comparison of cohorts	
	All (N=315)	Alive (N=281)	Dead (N=34)	*P* value	All (N=139)	Alive (N=118)	Dead (N=21)	*P* value	Effect size (Hodges–Lehmann)	*P* value
Demographics										
Age, y	76.0 (67.0 to 82.0)	76.0 (66.0 to 81.0)	76.0 (68.8 to 86.0)	0.236	77.3 (70.0 to 82.2)	76.4 (69.3 to 82.2)	79.0 (75.7 to 83.8)	0.092	1.00 (−1.04 to 3.11)	0.344
Sex (female)	136 (43.2%)	121 (43.1%)	15 (44.1%)	1.000	70 (50.4%)	57 (48.3%)	13 (61.9%)	0.344		0.184
Physical examination at the time of admission										
Body mass index, kg/m^2^	28.0 (25.0 to 31.6)	27.9 (25.0 to 31.2)	29.9 (26.2 to 35.1)	0.073	28.4 (24.7 to 32.0)	28.7 (25.4 to 32.2)	24.6 (22.7 to 31.2)	0.113	0.14 (−0.92 to 1.21)	0.772
Mean arterial pressure, mm Hg	100.0 (88.3 to 118.3)	101.7 (90.0 to 120.0)	90.0 (77.1 to 101.2)	<0.001*	103.3 (93.3 to 119.2)	103.3 (95.4 to 122.5)	86.7 (80.0 to 103.3)	<0.001*	1.67 (−3.33 to 6.67)	0.349
Heart rate, beats/min	100.0 (80.0 to 116.0)	100.0 (80.0 to 117.0)	100.0 (70.8 to 114.0)	0.376	100.0 (80.0 to 120.0)	102.0 (80.8 to 120.0)	90.0 (75.0 to 108.0)	0.088	1.00 (−4.00 to 7.00)	0.542
Respiratory rate, breaths/min	28.0 (24.0 to 33.0)	28.0 (24.0 to 32.0)	30.0 (26.0 to 34.8)	0.013*	28.0 (21.5 to 31.0)	28.0 (20.0 to 31.5)	30.0 (25.0 to 30.0)	0.219	−2.00 (−3.00 to 0.00)	0.084
Lung rales	311 (98.7%)	277 (98.6%)	34 (100.0%)	1.000	122 (87.8%)	102 (86.4%)	20 (95.2%)	0.469		<0.001*
Liver enlargement	176 (55.9%)	151 (53.7%)	25 (73.5%)	0.029*	49 (35.3%)	40 (33.9%)	9 (42.9%)	0.463		<0.001*
Ascites	49 (15.6%)	44 (15.7%)	5 (14.7%)	1.000	21 (15.1%)	19 (16.1%)	2 (9.5%)	0.740		1.000
Leg edema	204 (64.8%)	179 (63.7%)	25 (73.5%)	0.342	95 (68.3%)	79 (66.9%)	16 (76.2%)	0.457		0.520
Echocardiography										
Left ventricular ejection fraction, %	40.0 (30.0 to 50.0)	40.0 (30.0 to 50.0)	35.0 (25.0 to 46.5)	0.364	40.0 (35.0 to 50.0)	45.0 (35.0 to 50.0)	40.0 (30.0 to 48.8)	0.601	3.00 (0.00 to 5.00)	0.022*
Systolic pulmonary artery pressure, mm Hg	50.0 (45.0 to 60.0)	50.0 (45.0 to 55.0)	55.0 (50.0 to 60.0)	0.005*	45.0 (40.0 to 50.0)	45.0 (40.0 to 50.0)	50.0 (45.0 to 57.5)	0.102	−5.00 (−5.00 to −0.00)	0.010*
New York Heart Association class										
3 or 4	315 (100%)	281 (100%)	34 (100%)	…	128 (92.1%)	107 (90.7%)	21 (100.0%)	0.216		<0.001*
AHF type				1.000				0.022*		<0.001*
AHF following acute coronary syndrome	27 (8.6%)	24 (8.5%)	3 (8.8%)		43 (30.9%)	41 (34.7%)	2 (9.5%)			
Worsening of chronic heart failure	288 (91.4%)	257 (91.5%)	31 (91.2%)		96 (69.1%)	77 (65.3%)	19 (90.5%)			
Laboratory findings at the time of admission										
N‐terminal pro‐brain natriuretic peptide, pg/mL	6692.0 (3531.0 to 14395.5)	6112.0 (3386.0 to 12742.0)	13076.0 (6461.0 to 21952.2)	<0.001*	9532.0 (3643.0 to 17476.5)	7811.0 (3206.0 to 16933.0)	14936.0 (12544.0 to 20165.0)	<0.001*	882.00 (−568.00 to 2561.00)	0.254
Creatinine, μmol/L	117.0 (90.5 to 152.5)	114.0 (88.0 to 150.0)	133.5 (112.2 to 166.0)	0.008*	105.5 (88.0 to 136.8)	104.5 (88.2 to 134.8)	117.0 (79.8 to 143.2)	0.973	−8.00 (−16.00 to 0.00)	0.059
Estimated glomerular filtration rate, mL/min/1.73 m^2^	46.6 (32.3 to 65.0)	47.9 (32.7 to 67.1)	38.3 (29.5 to 49.6)	<0.001*	51.0 (32.6 to 66.9)	51.8 (32.6 to 67.3)	44.7 (33.6 to 64.3)	0.530	2.36 (−1.92 to 6.78)	0.283
Serum urea nitrogen, mmol/L	9.6 (6.9 to 14.4)	9.0 (6.8 to 13.6)	14.0 (11.2 to 18.1)	<0.001*	8.0 (6.0 to 13.0)	8.0 (6.0 to 11.0)	12.5 (7.0 to 15.0)	0.089	−0.90 (−1.70 to 0.00)	0.056
Sodium, mmol/L	140.0 (136.5 to 142.0)	140.0 (137.0 to 142.0)	137.0 (135.0 to 140.0)	0.010*	140.0 (138.0 to 143.0)	140.5 (138.0 to 143.0)	138.0 (136.0 to 141.0)	0.141	1.00 (−0.00 to 2.00)	0.071
Potassium, mmol/L	4.5 (4.1 to 4.8)	4.5 (4.1 to 4.8)	4.7 (4.0 to 5.1)	0.354	4.4 (4.0 to 4.7)	4.4 (4.0 to 4.7)	4.3 (3.7 to 4.9)	0.768	−0.10 (−0.20 to 0.00)	0.070
Chloride, mmol/L	103.0 (99.0 to 106.0)	103.0 (99.0 to 107.0)	98.5 (95.2 to 101.0)	<0.001*	104.0 (100.0 to 106.0)	104.0 (100.2 to 106.0)	102.0 (95.0 to 105.0)	0.128	0.00 (−1.00 to 1.00)	0.537
Aspartate aminotransferase, U/L	28.0 (20.0 to 44.5)	26.0 (20.0 to 42.0)	33.0 (21.5 to 161.2)	0.116	27.0 (20.8 to 38.0)	26.0 (20.0 to 36.2)	36.5 (26.2 to 55.5)	0.069	−0.00 (−3.00 to 2.00)	0.807
Alanine aminotransferase, U/L	25.0 (15.0 to 42.0)	24.0 (15.0 to 40.0)	28.5 (17.0 to 164.8)	0.250	23.0 (16.0 to 37.0)	23.0 (15.0 to 37.0)	23.0 (18.0 to 34.5)	0.524	−1.00 (−4.00 to 2.00)	0.357
Total protein, g/L	67.0 (61.0 to 72.0)	67.0 (61.0 to 72.0)	66.0 (61.0 to 73.0)	0.974	68.0 (63.0 to 72.0)	68.0 (63.0 to 73.0)	66.0 (60.0 to 70.5)	0.126	1.00 (−0.00 to 3.00)	0.074
Albumin, g/L	37.8 (34.8 to 41.3)	37.9 (35.0 to 41.5)	37.3 (34.7 to 39.4)	0.142	40.0 (35.0 to 50.2)	40.0 (36.0 to 52.0)	36.0 (32.5 to 41.5)	0.065	3.00 (1.30 to 4.80)	0.002*
Glucose, mmol/L	7.9 (6.1 to 11.2)	7.8 (6.0 to 11.0)	9.0 (6.8 to 11.7)	0.075	8.0 (6.2 to 11.0)	8.2 (6.4 to 11.0)	7.2 (5.7 to 10.0)	0.253	0.10 (−0.50 to 0.70)	0.704
Total cholesterol, mmol/L	3.5 (2.9 to 4.5)	3.6 (2.9 to 4.6)	3.0 (2.7 to 4.1)	0.019*	3.8 (3.2 to 4.8)	4.1 (3.3 to 5.0)	3.1 (2.8 to 4.3)	0.014*	0.35 (0.11 to 0.59)	0.003*
Low‐density lipoprotein cholesterol, mmol/L	1.9 (1.4 to 2.7)	1.9 (1.4 to 2.7)	1.6 (1.3 to 2.5)	0.090	2.3 (1.8 to 3.2)	2.4 (1.9 to 3.3)	1.9 (1.6 to 2.5)	0.016*	0.43 (0.24 to 0.61)	<0.001*
High‐density lipoprotein cholesterol, mmol/L	1.1 (0.9 to 1.3)	1.1 (0.9 to 1.4)	0.9 (0.7 to 1.2)	0.007*	1.0 (0.8 to 1.2)	1.0 (0.8 to 1.2)	0.8 (0.6 to 1.1)	0.218	−0.12 (−0.19 to −0.06)	<0.001*
Triglyceride, mmol/L	1.0 (0.8 to 1.3)	1.0 (0.8 to 1.3)	1.0 (0.8 to 1.3)	0.784	1.1 (0.9 to 1.4)	1.1 (0.9 to 1.4)	1.0 (0.8 to 1.2)	0.241	0.07 (0.01 to 0.15)	0.037*
C‐reactive protein, mg/L	12.2 (5.5 to 33.1)	11.4 (5.3 to 29.6)	30.4 (10.1 to 57.0)	0.004*	9.4 (3.7 to 27.0)	8.9 (3.0 to 21.8)	19.7 (5.7 to 41.7)	0.105	−2.00 (−4.20 to −0.00)	0.049*
Interleukin‐6, pg/mL	25.1 (12.9 to 60.1)	24.5 (12.4 to 54.3)	66.8 (22.2 to 133.0)	<0.001*	19.4 (8.8 to 43.9)	17.9 (8.5 to 38.1)	40.6 (24.3 to 130.3)	<0.001*	−4.60 (−9.00 to −0.70)	0.021*
Fibrinogen, g/L	4.0 (3.4 to 4.8)	4.0 (3.4 to 4.7)	4.0 (3.0 to 5.0)	0.976	3.6 (2.9 to 4.4)	3.6 (3.0 to 4.2)	3.8 (2.8 to 4.6)	0.698	−0.30 (−0.60 to −0.10)	0.002*
Platelets, ×10^12^/L	224.0 (181.0 to 277.0)	221.0 (181.0 to 272.0)	231.5 (188.2 to 316.2)	0.347	217.0 (155.5 to 266.5)	218.0 (160.0 to 266.8)	209.0 (148.0 to 262.0)	0.727	−10.00 (−26.00 to 7.00)	0.256
Hemoglobin, g/L	134.0 (119.0 to 148.0)	135.0 (120.0 to 148.0)	122.0 (109.5 to 139.5)	0.010*	136.0 (120.8 to 148.0)	136.0 (124.0 to 149.0)	129.0 (116.0 to 143.0)	0.217	2.00 (−2.00 to 6.00)	0.407
Leukocytes, ×10^9^/L	9.9 (7.5 to 13.1)	9.6 (7.4 to 13.1)	10.8 (8.0 to 13.3)	0.318	9.8 (7.8 to 12.8)	9.7 (7.7 to 12.8)	10.8 (8.8 to 12.6)	0.408	0.20 (−0.60 to 0.90)	0.673

Data are presented as n (%) or median and interquartile range (Q1, Q3). Differences between patients with AHF in the discovery and validation cohort were tested with the Brunner–Munzel test or Fischer’s exact test. Effect sizes are reported as Hodges–Lehmann estimates with corresponding 95% CIs. AHF indicates acute heart failure.

* *P* values <0.05, which are considered significant.

### Discovery of the Lipids Associated With In‐Hospital Mortality in Patients With AHF

**Figure 1:**
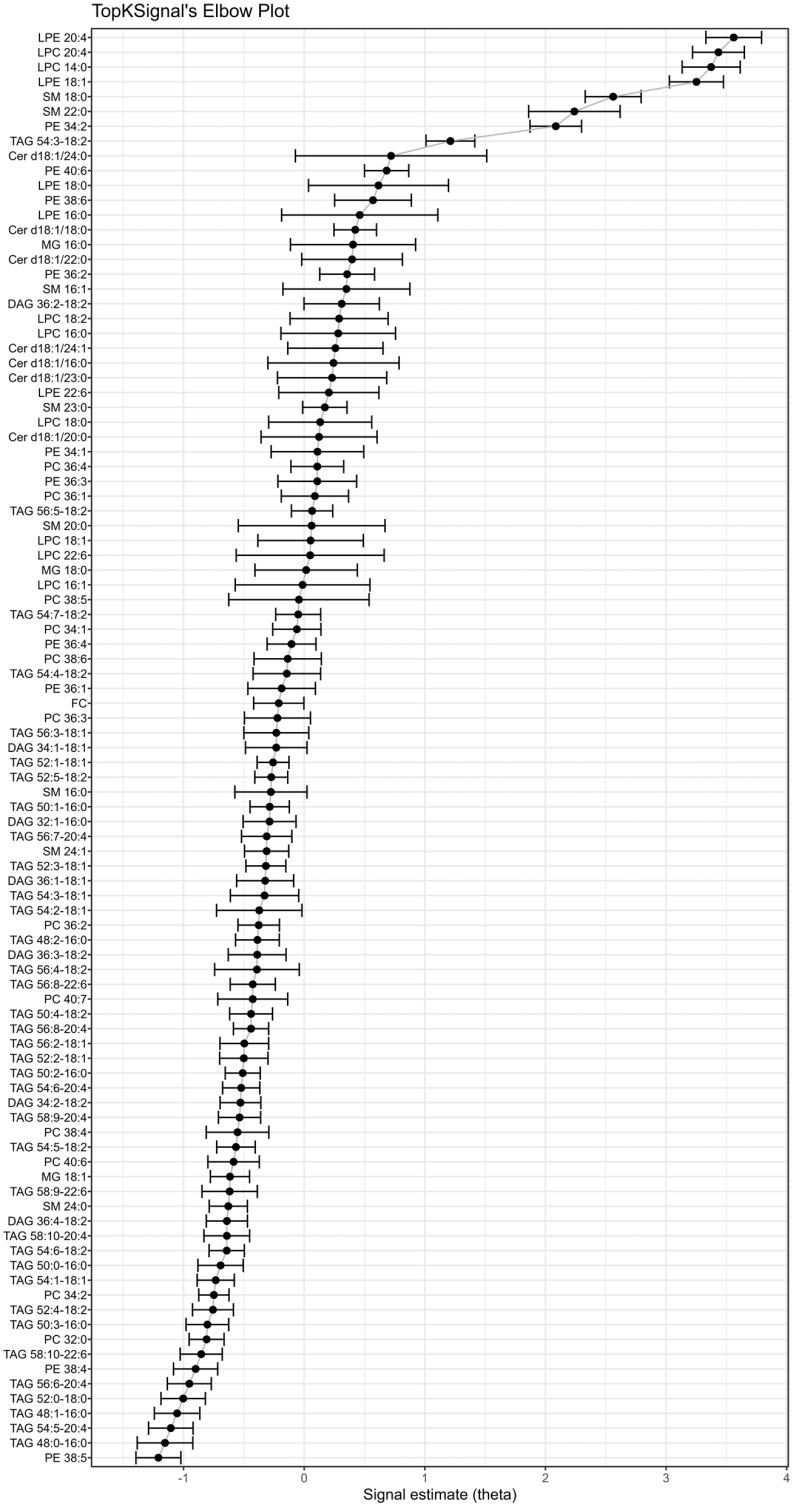
Results of TopKSignal’s displayed in an elbow plot. Horizontal bars around the signal estimate show SEs. Cer indicates ceramide; DAG, diacylglycerol; FC, free cholesterol; LPC, lysophosphatidylcholine; LPE, lysophosphatidylethanolamine; MG, monoacylglycerol; PC, phosphatidylcholine; PE, phosphatidylethanolamine; SM, sphingomyelin; and TAG, triacylglycerol.

Targeted lipidomics identified 117 serum lipids in the discovery cohort, of which 97 were included in orthogonal partial least squares discrimination analysis, least absolute shrinkage and selection operator regression, and random forest (Boruta) analyses (Table S1). Consolidation of these results using the TopKSignal ranking method identified lysophosphatidylethanolamine 20:4, lysophosphatidylcholine 20:4, lysophosphatidylcholine 14:0, and lysophosphatidylethanolamine 18:1 as the lipids most strongly associated with in‐hospital mortality (Figure 1). These lipids were selected because of nonoverlapping signal estimate standard errors compared with other candidates.

**Figure 2:**
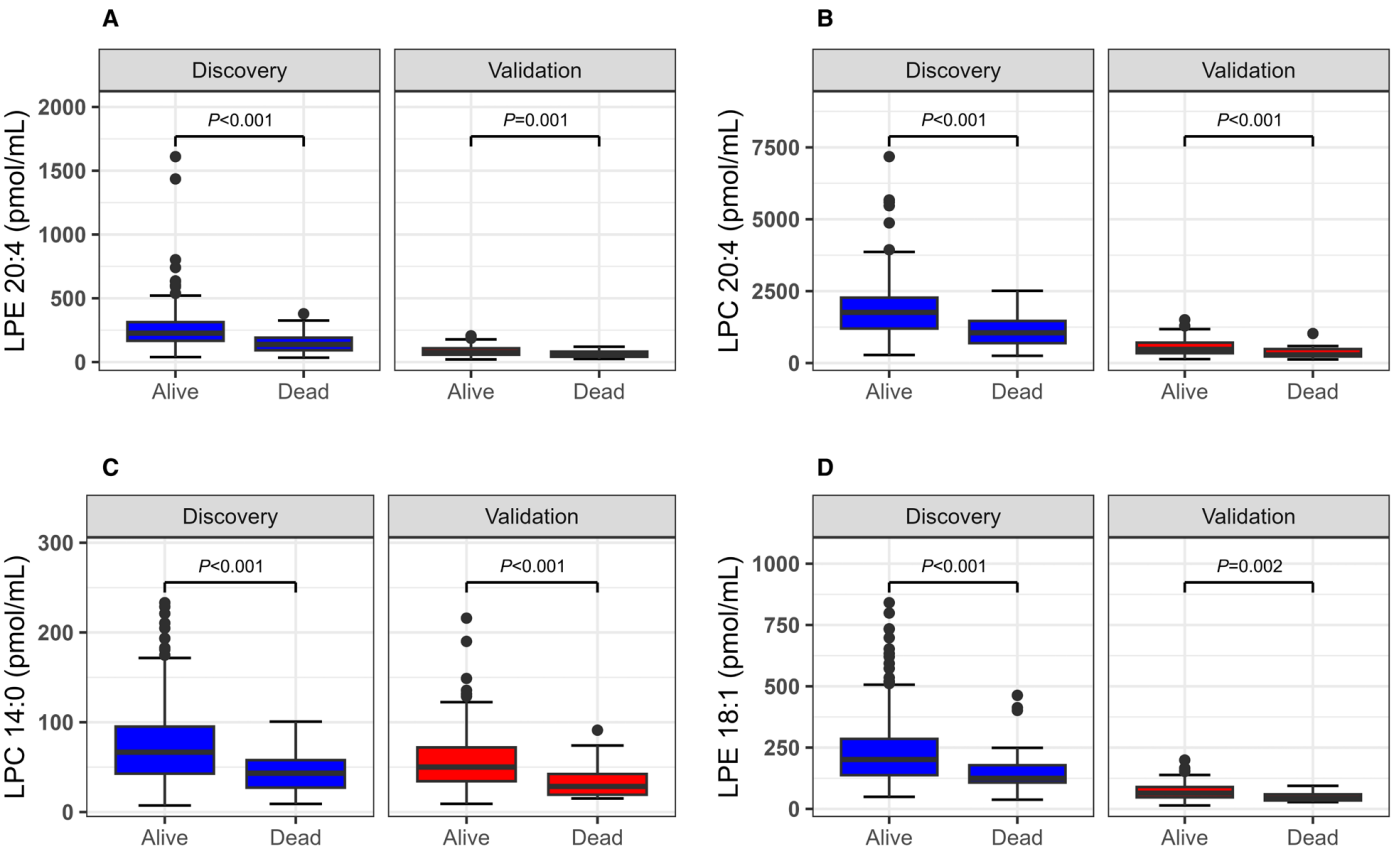
Comparison of (A) LPE 20:4, (B) LPC 20:4, (C) LPC 14:0, or (D) LPE 18:1 levels between patients with AHF who were alive and those who died in hospital. The lower and upper hinges of the box correspond to the first and third quartiles, whereas the line in the box corresponds to the median. The upper whisker extends from the hinge to the largest value no further than 1.5*IQR from the hinge (where IQR is the distance between the first and third quartiles). The lower whisker extends from the hinge to the smallest value at most 1.5*IQR of the hinge. Data beyond the end of the whiskers are considered outliers and plotted individually. Differences between the groups were tested with the Mann–Whitney *U* test. *P* values <0.05 are considered significant. Different responses of internal standards and different software used for the processing of the mass spectrometry data preclude a direct comparison of the absolute concentrations of lipids between discovery and validation cohort. AHF indicates acute heart failure; IQR, interquartile range; LPC, lysophosphatidylcholine; and LPE, lysophosphatidylethanolamine.

**Figure 3:**
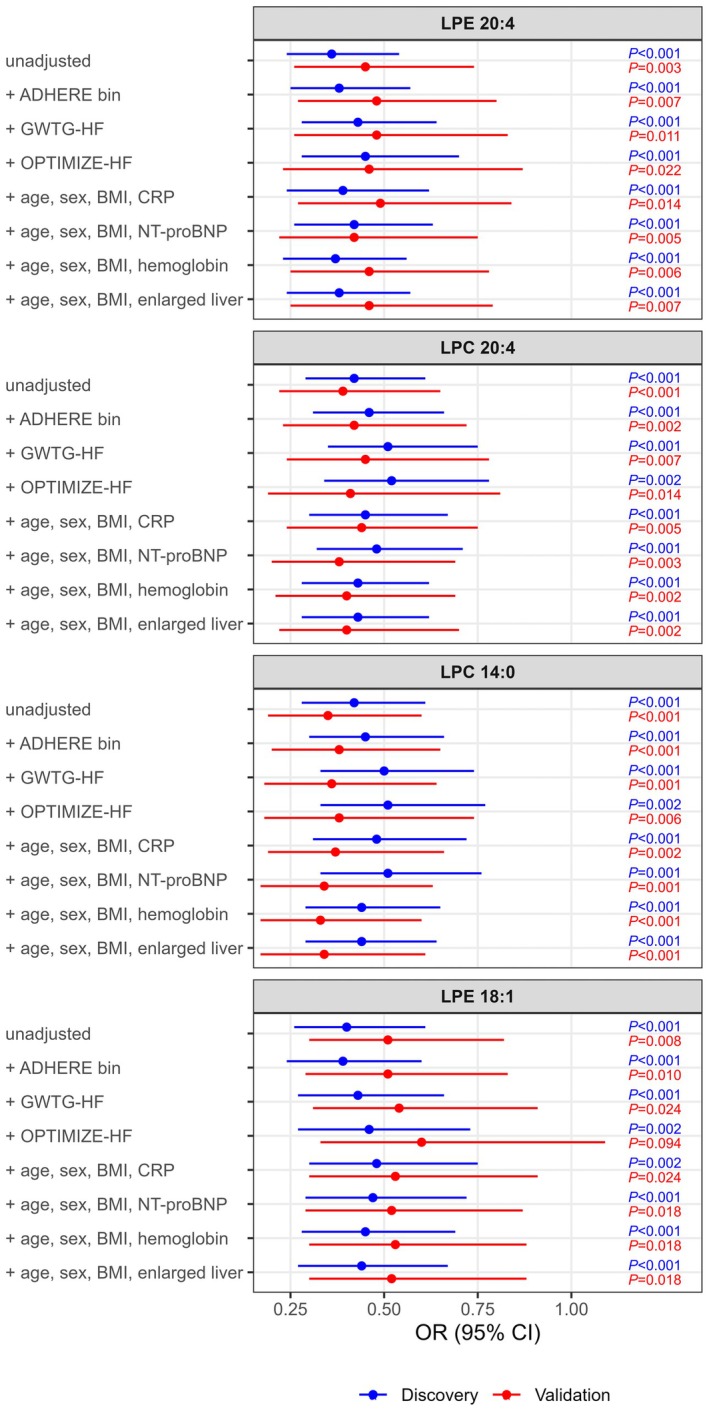
Univariable and adjusted logistic regression analyses of lipid parameters as predictors of in‐hospital mortality in the discovery and validation cohort of patients with AHF. *P* values <0.05 are considered significant. ADHERE indicates Acute Decompensated Heart Failure National Registry; AHF, acute heart failure; BMI, body mass index; CRP, C‐reactive protein; GWTG‐HF, Get With The Guidelines Heart Failure; HDL‐C, high‐density lipoprotein cholesterol; LPC, lysophosphatidylcholine; LPE, lysophosphatidylethanolamine; NT‐proBNP, N‐terminal pro brain natriuretic peptide; OPTIMIZE‐HF, Organized Program to Initiate Lifesaving Treatment in Hospitalized Patients With Heart Failure; and OR, odds ratio.

Serum concentrations of all 4 lipids were significantly lower in nonsurvivors than in survivors (Figure 2, Table S2). Univariable logistic regression confirmed strong inverse associations between lipid levels and in‐hospital mortality, which remained significant after adjustment for ADHERE, GWTG‐HF, or OPTIMIZE‐HF scores and other clinical and laboratory predictors of mortality, except for lysophosphatidylethanolamine 18:1 after adjustment for OPTIMIZE‐HF in the validation cohort (Figure 3, Table S3).

### Prognostic Ability and Clinical Utility of the Top 4 Selected Lipids

**Figure 4:**
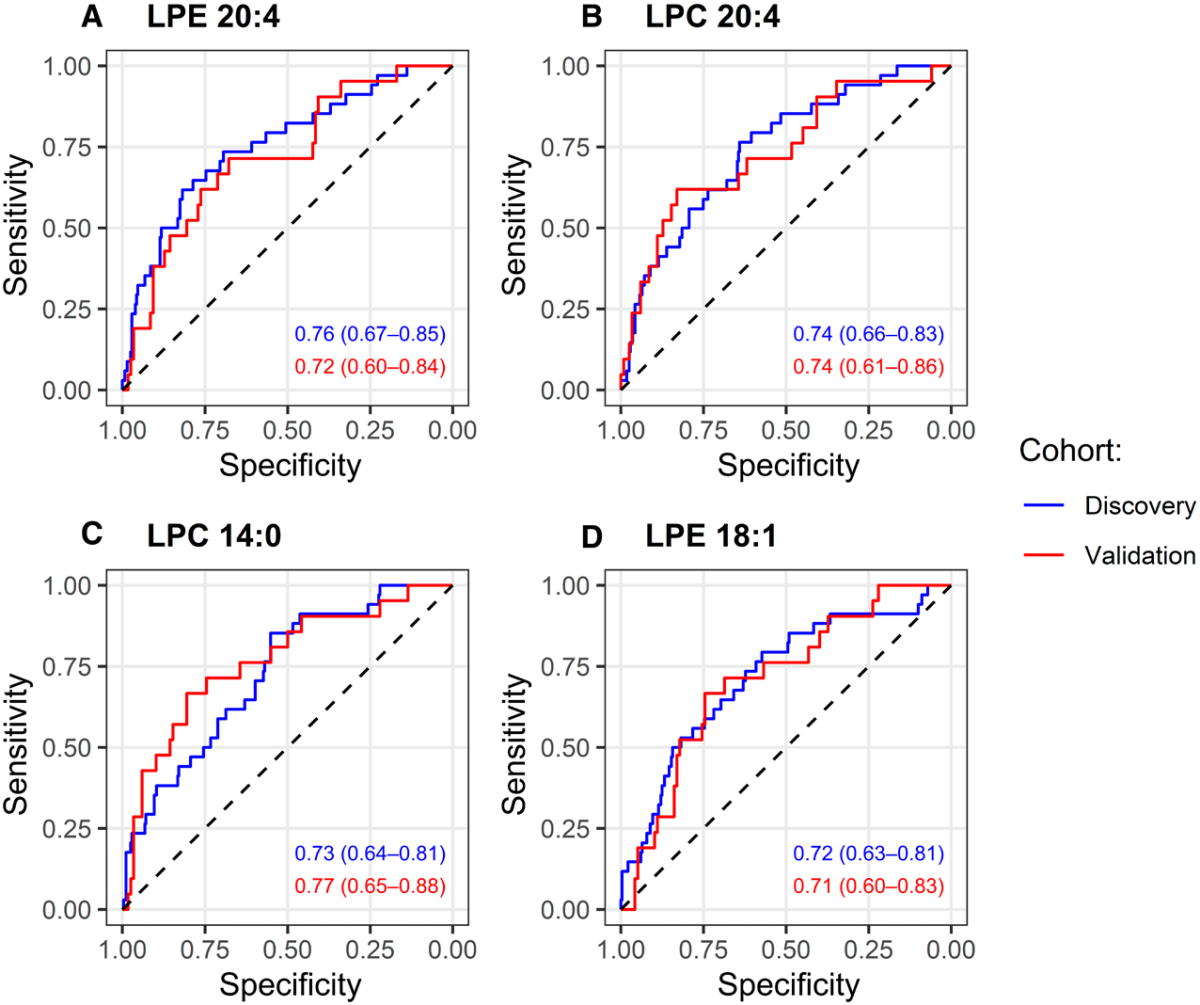
Receiver operating characteristic curves for prediction of in‐hospital mortality of (A) LPE 20:4, (B) LPC 20:4, (C) LPC 14:0, or (D) LPE 18:1. LPC indicates lysophosphatidylcholine; and LPE, lysophosphatidylethanolamine.

ROC curve analyses showed that in the discovery cohort, lysophosphatidylethanolamine 20:4 exhibited the highest prognostic accuracy, followed by lysophosphatidylcholine 20:4, lysophosphatidylcholine 14:0, and lysophosphatidylethanolamine 18:1. In the validation cohort, lysophosphatidylcholine 14:0 performed best, followed by lysophosphatidylcholine 20:4, lysophosphatidylethanolamine 20:4, and lysophosphatidylethanolamine 18:1 (Figure 4).

When added to established risk models, all 4 lipids significantly improved the AUC for ADHERE in both cohorts (Table 2). In the discovery cohort, lysophosphatidylethanolamine 20:4, lysophosphatidylcholine 20:4, and lysophosphatidylcholine 14:0 also significantly improved the AUC for GWTG‐HF, and lysophosphatidylcholine 14:0 further improved OPTIMIZE‐HF performance. Although similar trends were observed in the validation cohort, the AUC increases were not statistically significant.

**Table 2:** Discrimination Improvement by the Lipids for Risk Prediction of Hospital Mortality

	Discovery cohort (N=315)			Validation cohort (N=139)		
	AUC (95% CI)	ΔAUC (95% CI)	*P* value	AUC (95% CI)	ΔAUC (95% CI)	*P* value
Acute Decompensated Heart Failure National Registry	0.65 (0.56–0.74)	Reference		0.63 (0.51–0.74)	Reference	
+ Lysophosphatidylethanolamine 20:4	0.78 (0.69–0.87)	0.13* (0.05–0.21)*	0.002*	0.74 (0.64–0.85)	0.12* (0.02–0.22)*	0.024*
+ Lysophosphatidylcholine 20:4	0.77 (0.70–0.85)	0.12* (0.05–0.20)*	0.001*	0.75 (0.64–0.87)	0.12* (0.00–0.25)*	0.049*
+ Lysophosphatidylcholine 14:0	0.76 (0.68–0.85)	0.11* (0.04–0.18)*	0.002*	0.80 (0.71–0.89)	0.17* (0.04–0.30)*	0.011*
+ Lysophosphatidylethanolamine 18:1	0.76 (0.67–0.85)	0.12* (0.02–0.21)*	0.014*	0.76 (0.66–0.85)	0.13* (0.04–0.22)*	0.007*
Get With The Guidelines Heart Failure	0.70 (0.60–0.80)	Reference		0.74 (0.64–0.83)	Reference	
+ Lysophosphatidylethanolamine 20:4	0.78 (0.70–0.87)	0.09* (0.01–0.16)*	0.020*	0.78 (0.69–0.87)	0.04 (−0.03–0.11)	0.268
+ Lysophosphatidylcholine 20:4	0.77 (0.68–0.86)	0.07* (0.01–0.14)*	0.021*	0.79 (0.69–0.88)	0.05 (−0.05–0.14)	0.328
+ Lysophosphatidylcholine 14:0	0.77 (0.68–0.86)	0.07* (0.02–0.12)*	0.012*	0.82 (0.74–0.90)	0.08 (−0.03–0.19)	0.133
+ Lysophosphatidylethanolamine 18:1	0.77 (0.69–0.86)	0.07 (−0.01–0.15)	0.074	0.78 (0.69–0.87)	0.04 (−0.03–0.11)	0.219
Organized program to Initiate Lifesaving Treatment in Hospitalized Patients With Heart Failure	0.72 (0.62–0.81)	Reference		0.65 (0.50–0.80)	Reference	
+ Lysophosphatidylethanolamine 20:4	0.79 (0.69–0.88)	0.07 (0.00–0.14)	0.062	0.75 (0.64–0.86)	0.10 (−0.07–0.27)	0.252
+ Lysophosphatidylcholine 20:4	0.78 (0.69–0.87)	0.07 (0.00–0.13)	0.053	0.74 (0.60–0.89)	0.09 (−0.14–0.32)	0.433
+ Lysophosphatidylcholine 14:0	0.78 (0.69–0.87)	0.06* (0.01–0.11)*	0.029*	0.78 (0.65–0.91)	0.13 (−0.08–0.34)	0.223
+ Lysophosphatidylethanolamine 18:1	0.76 (0.67–0.86)	0.05 (−0.04–0.13)	0.284	0.72 (0.59–0.85)	0.07 (−0.09–0.22)	0.417

The ΔAUC indicates the difference between the AUC of the reference model and the model additionally including the indicated lipids. *P* values of the ΔAUCs were obtained by DeLong’s method. AUC, area under the curve.

* Significant ΔAUC and *P* values.

**Figure 5:**
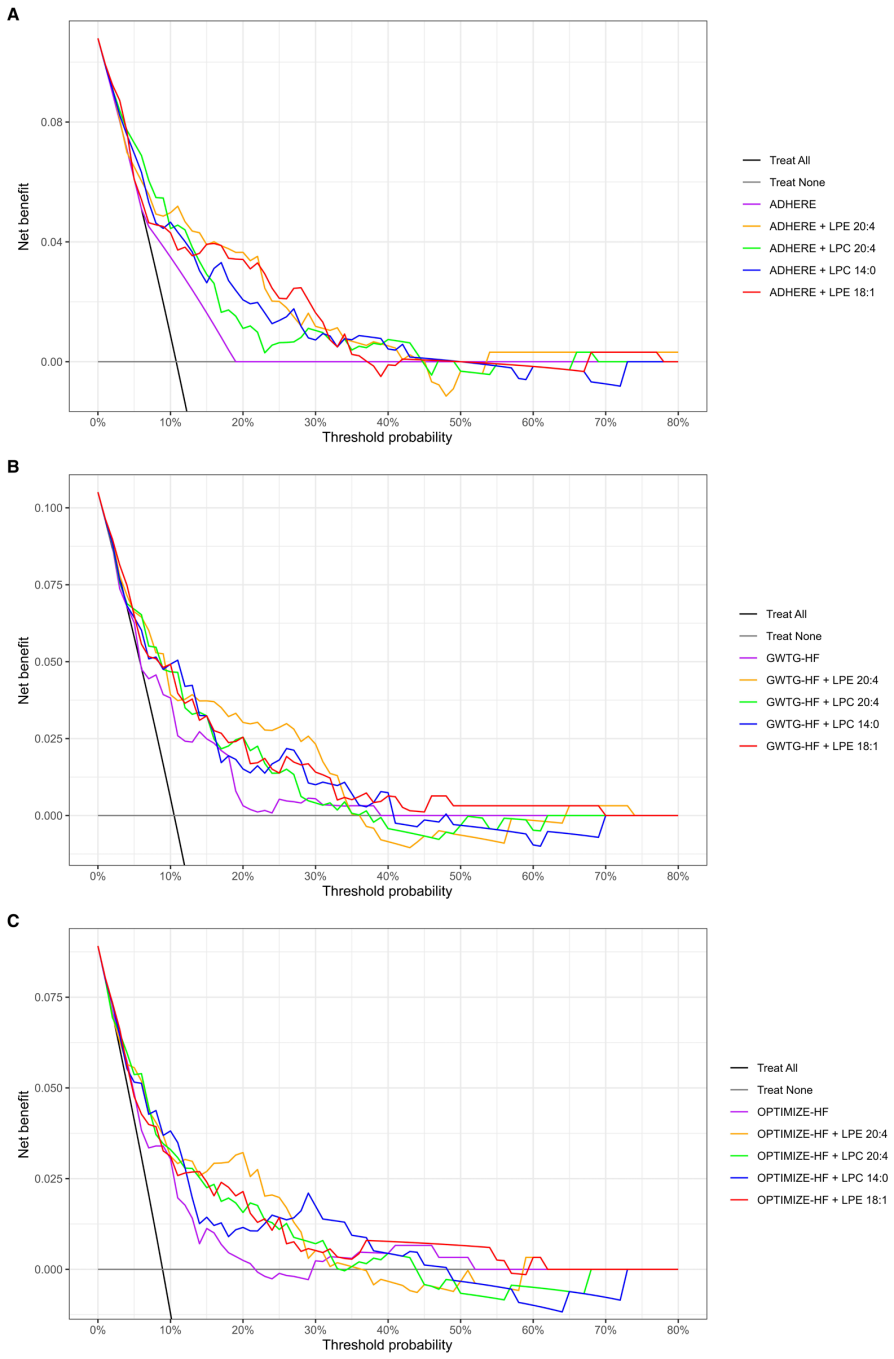
Decision curve analysis comparing net benefit of baseline risk scores—(A) ADHERE, (B) GWTG‐HF, or (C) OPTIMIZE‐HF—with lipid‐enhanced risk models for predicting in‐hospital mortality of patients with AHF in the discovery cohort. ADHERE indicates Acute Decompensated Heart Failure National Registry; AHF, acute heart failure; GWTG‐HF, Get With The Guidelines Heart Failure; LPC, lysophosphatidylcholine; LPE, lysophosphatidylethanolamine; and OPTIMIZE‐HF, Organized Program to Initiate Lifesaving Treatment in Hospitalized Patients With Heart Failure.

Decision curve analysis demonstrated that adding the individual lipids to established clinical scores increased the net clinical benefit for predicting in‐hospital mortality across threshold probabilities of ~12% to 35% in the discovery cohort (Figure 5A–C) and 15% to 28% in the validation cohort (Figure S1).

## DISCUSSION

In this study, we demonstrate for the first time that 4 lipids – lysophosphatidylethanolamine 20:4, lysophosphatidylcholine 20:4, lysophosphatidylcholine 14:0, and lysophosphatidylethanolamine 18:1, identified through serum lipidomics and comprehensive statistical approaches, improve the predictive performance of established clinical scores for in‐hospital mortality in patients with AHF. The inverse relationship between these lipids and AHF severity and mortality aligns with previous reports linking lower plasma lysophosphatidylcholines to adverse cardiovascular outcome.^33^, ^34^

Lysophosphatidylcholine and lysophosphatidylethanolamine are generated by cellular and serum phospholipases that cleave acyl chains from phosphatidylcholine and phosphatidylethanolamine, respectively.^35^, ^36^ Lysophosphatidylcholines are also produced during high‐density lipoprotein maturation by lecithin‐cholesterol acyltransferase and by reactive oxygen species in oxidized low‐density lipoprotein.^36^, ^37^ Serum lysophosphatidylcholine and lysophosphatidylethanolamine levels reflect a balance among tissue release, serum generation, uptake, and reacylation, with lysophospholipases further degrading or converting lysophosphatidylcholines into bioactive molecules such as lysophosphatidic acid.^37^

The prognostic lipids were inversely correlated with NT‐proBNP, blood urea nitrogen, systolic pulmonary artery pressure, and creatinine, established markers of HF severity,^38^, ^39^, ^40^ and positively associated with serum proteins, HDL and LDL cholesterol, mean arterial pressure, and hemoglobin, all negatively affected by HF pathophysiology^41^, ^42^ (Figure S2). These findings support an inverse association between the analyzed lipids and AHF severity.

Whether these lipids actively modulate HF pathophysiology or primarily reflect disease severity remains unclear. Lysophosphatidylcholines and lysophosphatidylethanolamines are bioactive, influencing vascular reactivity, endothelial function, immune modulation, oxidative stress, and cholesterol efflux.^35^, ^36^, ^37^ Lysophosphatidylcholine 20:4 regulates endothelial function,^43^ whereas lysophosphatidylethanolamine 20:4 and lysophosphatidylcholine 14:0 exert anti‐inflammatory effects.^44^, ^45^ In line with this observations, these lipids were inversely correlated with inflammatory markers (Figure S2). Arachidonic acid acyl chains of lysophosphatidylethanolamine 20:4 and lysophosphatidylcholine 20:4 are metabolized into bioactive molecules involved in cardiovascular and metabolic regulation.^46^ Consequently, reduced levels of these lipids may reflect diminished protective or compensatory mechanisms. Nevertheless, the observational design precludes causal inference, and these lipids likely serve as markers of disease severity rather than direct effectors of mortality.

Although the observed increases in AUC were modest, they may still be clinically meaningful in AHF, where even small gains in predictive accuracy can guide management decisions, including closer monitoring or intensified therapy. Consistent with prior work showing that ΔAUCs of ~0.02 to 0.19 can be meaningful,^22^, ^33^, ^47^ we observed ΔAUCs ranging from 0.04 to 0.17 after considering lipids on top of established clinical scores. The largest gains were seen in the ADHERE model, with statistically significant improvements in both cohorts. Smaller but directionally consistent enhancements were observed for the GWTG‐HF and OPTIMIZE‐HF models, remaining within the range generally considered clinically relevant for prognostic refinement.^33^, ^48^, ^49^, ^50^, ^51^ Favorable decision‐curve profiles, demonstrating a consistent net benefit across clinically relevant probability thresholds, further support the practical utility of integrating these lipids into existing prognostic tools. Our findings indicate that the identified lipids provide incremental prognostic value beyond conventional predictors. Accordingly, they may serve as early warning markers to identify high‐risk patients, enabling timely interventions such as inotrope initiation or adjustment, early consideration of mechanical circulatory support, or prioritization for heart transplantation. Given the risks and limitations of advanced therapies, optimizing timing and patient selection remains essential. Prospective studies are warranted to determine whether lipid‐guided management can improve outcomes and reduce mortality in hospitalized patients with AHF.

### Study Strengths and Limitations

Strengths include well‐characterized cohorts and standardized serum collection, storage, and processing. There are, however, several limitations: The study cohorts differed in some inherent features, such as the disease chronicity and severity, as well as the extent of congestion and inflammation. Additionally, the duration of sample storage before MS analysis differed between cohorts (4 versus 8 years). Spontaneous or lipases‐mediated degradation, as well as oxidation, can alter the concentrations of lipid species even when serum is stored at −80 °C.^52^, ^53^, ^54^, ^55^ Thus, although preanalytical handling was consistent across the study, long‐term storage may still have influenced lipid levels and their associations with mortality.

Lipidomics data represent a single admission snapshot, precluding analysis of lipid dynamics. Because the patients’ nutritional status at admission was unknown, we could not assess the potential confounding effects of fed versus fasted states on the associations between the studied lipids and mortality. Because all patients were enrolled in a single center, the generalizability of the present data is limited. Despite its better sensitivity and selectivity, the limited lipid coverage of the targeted lipidomics approach might have precluded identification of additional potential prognostic lipids. The rather moderate sample sizes, particularly the small number of deaths in the validation cohort, may have limited the robustness of our conclusions. The lack of significance in assessing the impact of lipids on discrimination improvement in the validation cohort may have been due to the limited number of events. Therefore, additional larger studies are needed to confirm the presented results.

## CONCLUSIONS

The identified lipids demonstrated moderate predictive ability for in‐hospital mortality in patients with AHF. Adding the individual lipids to established risk scores improved discrimination. Their prognostic value likely reflects multifactorial regulation and diverse bioactivities. Overall, these lipids are promising biomarkers whose clinical utility should be validated in larger cohorts with AHF.

## Sources of Funding

Tobias Madl is grateful to the Austrian Science Fund for excellence cluster 10.55776/COE14, Grants DOIs 10.55776/P28854, 10.55776/I3792, 10.55776/DOC130, and 10.55776/W1226, the Austrian Research Promotion Agency grants 864690 and 870454, the Integrative Metabolism Research Center Graz, the Austrian Infrastructure Program 2016/2017, the Styrian Government, the City of Graz and BioTechMed‐Graz. This work was also funded in part by the Austrian Research Promotion Agency and the European Union under the grant 912192. The funders had no role in study design; in the collection, analysis, and interpretation of data; as well as in the writing of the report and in the decision to submit the article for publication. For open access purposes, the author has applied a CC BY public copyright license to any author accepted article version arising from this submission.

## Disclosures

None.

## Supporting information

Supplemental Methods S1.

Tables S1–S3
Figures S1–S2
