# Supplementary material for: Low Serum Lysophospholipids Predict Increased In‐Hospital Mortality in Patients With Acute Heart Failure
Source: J Am Heart Assoc. 2026 Jan 19;15(2):e043828. doi: 10.1161/JAHA.125.043828 (PMC12919526; doi:10.1161/JAHA.125.043828)
Supplement: Supplementary file 1 — Supplemental Methods S1. [file JAH3-15-e043828-s001.pdf]

SUPPLEMENTAL METHODS and RESULTS

Acquisition Method Report

Chemicals

	MTBE	Roth
	Isopropanaol	Sigma
	water	double destilled, in- house
	Methanol	ChemLab
	formic acid	Sigma
	phosphoric acid	Sigma
	Ammoniumacetate	Sigma

Internal standards (IS)			Stock solution [µM] in chloroform/methanol 2/1 v/v
	TAG 54:9	Larodan	12,2
	TAG 51:0	Larodan	58,6
	TAG 45:0	Larodan	10,7
	DAG 34:0	Larodan	15,9
	DAG 28:0	Avanti	13,7
	FC d7	Avanti	330,6
	MG 17:0	Larodan	36,7
	PC 28:0	Larodan	94,9
	PE 34:0	Avanti	268,8
	LPC 17:1	Avanti	20,3
	LPE 17:1	Avanti	139,7
	Cer 35:0	Avanti	7,4
	SM 17:0	Avanti	71,7

Device List

	Multisampler	Model:	G7167B	Agilent
	Binary Pump	Model:	G7120A	Agilent
	Column Comp.	Model:	G7116B	Agilent
	QQQ	Model:	6470	Agilent

**Name:**	**Multisampler**	**Model:**	**G7167B**
Sampling Speed			
	Draw Speed	100.0 µL/min	
	Eject Speed	400.0 µL/min	
	Wait Time After Drawing	1.2 s	
Injection			
	Injection Volume	2.00 µL	
	Needle Wash Mode	Multi-wash	
	Sample Flush-Out Factor	5.0	
	Draw Position Offset	2.0 mm	
	Use Vial/Well Bottom Sensing	Yes	

**Name:**	**Binary Pump**	**Model:**	**G7120A**
Pump Parameters			
	Flow	0.150 mL/min	
	Low Pressure Limit	0.00 bar	
	High Pressure Limit	880.00 bar	
	Stoptime	30.00 min	
Solvent Composition			
	Channel		

	A	100.0 % Water, 0.1 % Formic Acid, 1 mM NH4Ac, 8 µM H3PO4	
	B	100.0 % Isopropanol, 0.1 % Formic Acid, 1 mM NH4Ac, 8 µM H3PO4	
Timetable			
	Time	A	B
	0.30 min	50.00 %	50.00 %
	0.50 min	50.00 %	50.00 %
	9.00 min	20.00 %	80.00 %
	22.00 min	0.00 %	100.00 %
	24.50 min	0.00 %	100.00 %
	25.00 min	50.00 %	50.00 %
	29.70 min	50.00 %	50.00 %
	30.00 min	50.00 %	50.00 %
**Name:**	**Column Comp.**	**Model:**	**G7116B**
	Temperature Control		
	Mode	Temperature Set	
	Temperature	50.0 °C	
**Name:**	**MS QQQ Mass Spectrometer**	**Model:**	**6470**
Source Parameters			
	Ion Source	AJS ESI	
	Gas:	Nitrogen	
		Value (+)	
	Gas Temp (°C)	300	

Gas Flow (l/min)	5
Nebulizer (psi)	30
SheathGasHeater	400
SheathGasFlow	12
Capillary (V)	3500
VCharging	0

Scan Parameters

Scan Type	DynamicMRM
Ion Mode	ESI+Agilent Jet Stream
Cycle Time (ms)	700
Data STG	Centroid

MRM parameters

Scan Segments

Cpd Group	Cpd Name	Prec Ion	MS1 Res	Prod Ion	MS2 Res	Frag (V)	CE (V)	Cell Acc (V)	Ret Time (min)	Ret Window	Polarity
SL	CE	369.2	Unit	369.2	Unit	135	0	5	22	9	Positive
Cer	Cer d18:1/16:0_H2O	520.5	Unit	264.2	Unit	199	24	5	14.7	9	Positive
Cer	Cer d18:1/18:0_H2O	548.5	Unit	264.2	Unit	199	24	5	15.7	9	Positive
Cer	Cer d18:1/20:0_H2O	576.6	Unit	264.2	Unit	199	24	5	16.6	9	Positive
Cer	Cer d18:1/22:0_H2O	604.6	Unit	264.2	Unit	199	24	5	17.5	9	Positive
Cer	Cer d18:1/23:0_H2O	618.6	Unit	264.2	Unit	199	24	5	18	9	Positive
Cer	Cer d18:1/24:0_H2O	632.6	Unit	264.2	Unit	199	24	5	18.3	9	Positive
Cer	Cer d18:1/24:1_H2O	630.6	Unit	264.2	Unit	199	24	5	17.5	9	Positive
DAG	DAG 32:0-16:0	586.5	Unit	313	Unit	110	16	5	16.5	9	Positive
DAG	DAG 32:1-16:0	584.5	Unit	313	Unit	110	16	5	15.7	9	Positive

DAG	DAG 34:0-18:0	614.6	Unit	341	Unit	110	16	5	17.4	9	Positive
DAG	DAG 34:1-18:1	612.6	Unit	339	Unit	110	16	5	16.6	9	Positive
DAG	DAG 34:2-18:2	610.6	Unit	337	Unit	110	16	5	15.9	9	Positive
DAG	DAG 36:1-18:1	640.6	Unit	339	Unit	110	16	5	17.6	9	Positive
DAG	DAG 36:2-18:2	638.6	Unit	339	Unit	110	16	5	16.7	9	Positive
DAG	DAG 36:3-18:2	636.6	Unit	337	Unit	110	16	5	16	9	Positive
DAG	DAG 36:4-18:2	634.6	Unit	337	Unit	110	16	5	15.4	9	Positive
SL	FC	369.2	Unit	369.2	Unit	135	0	5	13.7	9	Positive
IS	IS FC d7	376.2	Unit	376.2	Unit	135	0	5	13.7	9	Positive
IS	IS LPE 17:1	479.3	Unit	325.2	Unit	111	16	5	7.6	9	Positive
IS	IS Cer35:1;2	552.5	Unit	264.2	Unit	199	24	5	15.15	9	Positive
IS	IS Cer35:1;2_H2O	534.5	Unit	264.2	Unit	199	24	5	15.15	9	Positive
IS	IS DAG 28:0-14:0	530.5	Unit	285.2	Unit	121	16	5	14.4	9	Positive
IS	IS DAG 34:0-17:0	614.6	Unit	327.3	Unit	137	20	5	17.4	9	Positive
IS	IS LPC 17:0	510.3	Unit	184.1	Unit	179	28	5	8	9	Positive
IS	IS LPC 17:1	508.3	Unit	184.1	Unit	79	28	5	7	9	Positive
IS	IS LPG 17:1	466.3	Unit	325.2	Unit	111	16	5	7.1	9	Positive
IS	IS MG 17:0	345.3	Unit	253.2	Unit	116	8	2	10	9	Positive
IS	IS PC 28:0	678.5	Unit	184.1	Unit	164	28	5	12.5	9	Positive
IS	IS PC 34:0	762.6	Unit	184.1	Unit	204	32	5	15.4	9	Positive
IS	IS PE 34:0	720.6	Unit	579.5	Unit	131	20	5	15.6	9	Positive
IS	IS SM 17:0	717.6	Unit	184.1	Unit	184	24	5	13.8	9	Positive
IS	IS TAG 45:0	782.7	Unit	523.5	Unit	169	24	5	21	9	Positive
IS	IS TAG 51:0	866.8	Unit	579.5	Unit	179	24	5	22.6	9	Positive
IS	IS TAG 54:9	890.9	Unit	595.6	Unit	189	24	5	19.7	9	Positive
LPC	LPC 14:0	468.3	Unit	184.1	Unit	179	28	5	5.9	9	Positive
LPC	LPC 16:0	496.3	Unit	184.1	Unit	179	28	5	7.4	9	Positive
LPC	LPC 16:1	494.3	Unit	184.1	Unit	179	28	5	6.3	9	Positive
LPC	LPC 18:0	524.3	Unit	184.1	Unit	179	28	5	8.8	9	Positive
LPC	LPC 18:1	522.3	Unit	184.1	Unit	179	28	5	7.7	9	Positive
LPC	LPC 18:2	520.3	Unit	184.1	Unit	179	28	5	6.8	9	Positive
LPC	LPC 20:4	544.3	Unit	184.1	Unit	179	28	5	6.9	9	Positive

LPC	LPC 22:6	568.3	Unit	184.1	Unit	179	28	5	6.8	9	Positive
LPE	LPE 16:0	454.3	Unit	313.3	Unit	111	16	5	7.6	9	Positive
LPE	LPE 18:0	482.3	Unit	341.3	Unit	111	16	5	8.9	9	Positive
LPE	LPE 18:1	480.3	Unit	339.3	Unit	111	16	5	7.9	9	Positive
LPE	LPE 20:4	502.3	Unit	361.3	Unit	111	16	5	7	9	Positive
LPE	LPE 22:6	526.3	Unit	385.3	Unit	111	16	5	7	9	Positive
MG	MG 16:0	331.3	Unit	239.2	Unit	116	8	2	9.2	9	Positive
MG	MG 16:1	329.3	Unit	237.2	Unit	116	8	2	8.1	9	Positive
MG	MG 18:0	359.3	Unit	267.3	Unit	116	8	2	10.6	9	Positive
MG	MG 18:1	357.3	Unit	265.3	Unit	116	8	2	9.5	9	Positive
MG	MG 18:2	355.3	Unit	263.2	Unit	116	8	2	8.7	9	Positive
MG	MG 20:4	379.3	Unit	287.2	Unit	116	12	2	8.7	9	Positive
MG	MG 22:6	403.3	Unit	311.3	Unit	116	12	2	8.7	9	Positive
PC	PC 32:0	734.6	Unit	184.1	Unit	180	30	5	14.4	9	Positive
PC	PC 32:1	732.6	Unit	184.1	Unit	180	30	5	13.7	9	Positive
PC	PC 34:1	760.6	Unit	184.1	Unit	180	30	5	14.6	9	Positive
PC	PC 34:2	758.6	Unit	184.1	Unit	180	30	5	13.9	9	Positive
PC	PC 36:0	790.6	Unit	184.1	Unit	180	30	5	16.3	9	Positive
PC	PC 36:1	788.6	Unit	184.1	Unit	180	30	5	15.5	9	Positive
PC	PC 36:2	786.6	Unit	184.1	Unit	180	30	5	14.9	9	Positive
PC	PC 36:3	784.6	Unit	184.1	Unit	180	30	5	14.3	9	Positive
PC	PC 36:4	782.6	Unit	184.1	Unit	180	30	5	13.9	9	Positive
PC	PC 38:3	812.6	Unit	184.1	Unit	180	30	5	15.2	9	Positive
PC	PC 38:4	810.6	Unit	184.1	Unit	180	30	5	14.9	9	Positive
PC	PC 38:5	808.6	Unit	184.1	Unit	180	30	5	14	9	Positive
PC	PC 38:6	806.6	Unit	184.1	Unit	180	30	5	13.8	9	Positive
PC	PC 40:6	834.6	Unit	184.1	Unit	180	30	5	14.8	9	Positive
PC	PC 40:7	832.6	Unit	184.1	Unit	180	30	5	13.8	9	Positive
PE	PE 32:0	692.5	Unit	551.5	Unit	131	20	5	14.6	9	Positive
PE	PE 32:1	690.5	Unit	549.5	Unit	131	20	5	13.8	9	Positive
PE	PE 34:1	718.5	Unit	577.5	Unit	131	20	5	14.8	9	Positive
PE	PE 34:2	716.5	Unit	575.5	Unit	131	20	5	14.1	9	Positive

PE	PE 36:1	746.6	Unit	605.6	Unit	131	20	5	15.7	9	Positive
PE	PE 36:2	744.6	Unit	603.6	Unit	131	20	5	15.2	9	Positive
PE	PE 36:3	742.6	Unit	601.6	Unit	131	20	5	14.2	9	Positive
PE	PE 36:4	740.6	Unit	599.6	Unit	131	20	5	14.1	9	Positive
PE	PE 38:4	768.6	Unit	627.6	Unit	131	20	5	15.1	9	Positive
PE	PE 38:5	766.6	Unit	625.6	Unit	131	20	5	14.3	9	Positive
PE	PE 38:6	764.6	Unit	623.6	Unit	131	20	5	14	9	Positive
PE	PE 40:6	792.6	Unit	651.6	Unit	131	20	5	15	9	Positive
SM	SM 16:0	703.6	Unit	184.1	Unit	184	24	5	13.4	9	Positive
SM	SM 16:1	701.6	Unit	184.1	Unit	184	24	5	12.6	9	Positive
SM	SM 18:0	731.6	Unit	184.1	Unit	184	24	5	14.4	9	Positive
SM	SM 20:0	759.6	Unit	184.1	Unit	184	24	5	15.3	9	Positive
SM	SM 22:0	787.7	Unit	184.1	Unit	184	24	5	16.3	9	Positive
SM	SM 23:0	801.7	Unit	184.1	Unit	184	24	5	16.7	9	Positive
SM	SM 24:0	815.7	Unit	184.1	Unit	184	24	5	17.2	9	Positive
SM	SM 24:1	813.7	Unit	184.1	Unit	184	24	5	16.3	9	Positive
TAG	TAG 48:0-16:0	824.6	Unit	551.3	Unit	179	24	5	21.9	9	Positive
TAG	TAG 48:1-16:0	822.6	Unit	549.3	Unit	179	24	5	21.3	9	Positive
TAG	TAG 48:2-16:0	820.6	Unit	547.3	Unit	179	24	5	20.9	9	Positive
TAG	TAG 50:0-16:0	852.6	Unit	579.3	Unit	179	24	5	22.3	9	Positive
TAG	TAG 50:1-16:0	850.6	Unit	577.3	Unit	179	24	5	21.9	9	Positive
TAG	TAG 50:2-16:0	848.6	Unit	575.3	Unit	179	24	5	21.5	9	Positive
TAG	TAG 50:3-16:0	846.6	Unit	573.3	Unit	179	24	5	21	9	Positive
TAG	TAG 50:4-18:2	844.6	Unit	547.3	Unit	179	24	5	20.6	9	Positive
TAG	TAG 52:0-18:0	880.7	Unit	579.3	Unit	179	24	5	22.8	9	Positive
TAG	TAG 52:1-18:1	878.7	Unit	579.3	Unit	179	24	5	22.4	9	Positive
TAG	TAG 52:2-18:1	876.7	Unit	577.3	Unit	179	24	5	22	9	Positive
TAG	TAG 52:3-18:1	874.7	Unit	575.3	Unit	179	24	5	21.6	9	Positive
TAG	TAG 52:4-18:2	872.7	Unit	575.3	Unit	179	24	5	21.15	9	Positive
TAG	TAG 52:5-18:2	870.6	Unit	573.3	Unit	179	24	5	20.6	9	Positive
TAG	TAG 54:0-18:0	908.7	Unit	607.3	Unit	179	24	5	23.2	9	Positive
TAG	TAG 54:1-18:1	906.7	Unit	607.3	Unit	179	24	5	22.8	9	Positive

TAG	TAG 54:2-18:1	904.7	Unit	605.3	Unit	179	24	5	22.4	9	Positive
TAG	TAG 54:3-18:1	902.7	Unit	603.4	Unit	179	24	5	22	9	Positive
TAG	TAG 54:3-18:2	902.7	Unit	605.4	Unit	179	24	5	22	9	Positive
TAG	TAG 54:4-18:2	900.6	Unit	603.4	Unit	179	24	5	21.6	9	Positive
TAG	TAG 54:5-18:2	898.6	Unit	601.4	Unit	179	24	5	21.2	9	Positive
TAG	TAG 54:5-20:4	898.6	Unit	577.5	Unit	179	24	5	21.5	9	Positive
TAG	TAG 54:6-18:2	896.6	Unit	599.4	Unit	179	24	5	20.8	9	Positive
TAG	TAG 54:6-20:4	896.6	Unit	575.4	Unit	179	24	5	20.6	9	Positive
TAG	TAG 54:7-18:2	894.6	Unit	597.4	Unit	179	24	5	20.4	9	Positive
TAG	TAG 54:7-20:4	894.6	Unit	573.4	Unit	179	24	5	20.4	9	Positive
TAG	TAG 56:1-18:1	934.7	Unit	635.3	Unit	179	24	5	23.3	9	Positive
TAG	TAG 56:2-18:1	932.7	Unit	633.3	Unit	179	24	5	22.8	9	Positive
TAG	TAG 56:3-18:1	930.7	Unit	631.3	Unit	179	24	5	22.5	9	Positive
TAG	TAG 56:4-18:2	928.7	Unit	631.3	Unit	179	24	5	22.1	9	Positive
TAG	TAG 56:5-18:2	926.7	Unit	629.4	Unit	179	24	5	21.7	9	Positive
TAG	TAG 56:6-20:4	924.7	Unit	603.4	Unit	179	24	5	21.6	9	Positive
TAG	TAG 56:7-20:4	922.7	Unit	601.5	Unit	179	24	5	21.1	9	Positive
TAG	TAG 56:8-20:4	920.7	Unit	599.3	Unit	179	24	5	20.7	9	Positive
TAG	TAG 56:8-22:6	920.7	Unit	575.3	Unit	179	24	5	20.5	9	Positive
TAG	TAG 58:10-20:4	944.6	Unit	623.3	Unit	179	24	5	20.8	10	Positive
TAG	TAG 58:10-22:6	944.6	Unit	599.3	Unit	179	24	5	20.8	10	Positive
TAG	TAG 58:4-18:2	956.6	Unit	659.3	Unit	179	24	5	22.8	10	Positive
TAG	TAG 58:5-20:4	954.6	Unit	633.3	Unit	179	24	5	22.6	10	Positive
TAG	TAG 58:6-20:4	952.6	Unit	631.3	Unit	179	24	5	22	10	Positive
TAG	TAG 58:7-20:4	950.6	Unit	629.3	Unit	179	24	5	21.6	10	Positive
TAG	TAG 58:8-20:4	948.6	Unit	627.3	Unit	179	24	5	21.5	10	Positive
TAG	TAG 58:8-22:6	948.6	Unit	603.5	Unit	179	24	5	21.5	10	Positive
TAG	TAG 58:9-20:4	946.6	Unit	625.3	Unit	179	24	5	21.1	10	Positive
TAG	TAG 58:9-22:6	946.6	Unit	601.3	Unit	179	24	5	21	10	Positive

Cer, ceramide; DAG, diacylglycerol; FC, free cholesterol; IS, internal standard; LPC, lysophosphatidylcholine; LPE, lysophosphatidylethanolamine; MG, monoacylglycerol; OPLS-DA, orthogonal partial least squares discriminant analysis; PC, phosphatidylcholine; PE, phosphatidylethanolamine; SM, sphingomyelin; TAG, triacylglycerol.

Supplemental Table S1. Ranking of Lipids According to the Strength of Their Association With In-Hospital Mortality in AHF (Results of LASSO Regression, OPLS-DA or Random Forest (Boruta) Analyses).

Lipid	Lasso (n)	OPLS-DA (VIP score)	Boruta confirmed/tentative (n/n)
SM 16:0	0	0.735	0/0
SM 16:1	0	0.906	0/6
SM 18:0	139	1.122	0/3
SM 20:0	0	1.501	0/0
SM 22:0	2	1.144	0/0
SM 23:0	0	1.041	0/0
SM 24:0	0	0.905	0/0
SM 24:1	0	0.638	0/0
Cer d18:1/16:0	0	0.518	0/9
Cer d18:1/18:0	0	0.284	0/1
Cer d18:1/20:0	0	0.655	0/0
Cer d18:1/22:0	0	1.024	0/0
Cer d18:1/23:0	0	1.211	0/1
Cer d18:1/24:0	0	1.275	1/0
Cer d18:1/24:1	0	0.703	0/0
MG 16:0	0	0.635	0/0
MG 18:0	2	0.450	0/0
MG 18:1	0	0.917	0/0
DAG 32:1-16:0	0	0.666	0/0
DAG 34:1-18:1	0	0.603	0/0
DAG 34:2-18:2	0	0.576	0/0
DAG 36:1-18:1	0	0.438	0/0
DAG 36:2-18:2	0	0.602	0/0
DAG 36:3-18:2	0	0.582	0/0
DAG 36:4-18:2	0	0.425	0/0
TAG 48:0-16:0	204	0.003	3/7

TAG 48:1-16:0	0	0.016	0/0
TAG 48:2-16:0	0	0.119	0/0
TAG 50:0-16:0	0	0.050	0/1
TAG 50:1-16:0	0	0.196	0/0
TAG 50:2-16:0	0	0.131	0/0
TAG 50:3-16:0	0	0.044	0/0
TAG 50:4-18:2	0	0.113	0/0
TAG 52:0-18:0	0	0.021	0/0
TAG 52:1-18:1	0	0.165	0/0
TAG 52:2-18:1	0	0.155	0/0
TAG 52:3-18:1	0	0.137	0/0
TAG 52:4-18:2	0	0.046	0/0
TAG 52:5-18:2	0	0.192	0/0
TAG 54:1-18:1	0	0.236	0/0
TAG 54:2-18:1	0	0.362	0/0
TAG 54:3-18:1	0	0.276	0/0
TAG 54:3-18:2	5	0.417	0/2
TAG 54:4-18:2	0	0.255	0/1
TAG 54:5-18:2	0	0.080	0/0
TAG 54:5-20:4	0	0.005	0/0
TAG 54:6-18:2	0	0.074	0/0
TAG 54:6-20:4	0	0.081	0/0
TAG 54:7-18:2	0	0.399	0/0
TAG 56:2-18:1	0	0.239	0/0
TAG 56:3-18:1	0	0.359	0/0
TAG 56:4-18:2	0	0.337	0/1
TAG 56:5-18:2	0	0.220	0/0
TAG 56:6-20:4	0	0.025	0/0
TAG 56:7-20:4	0	0.170	0/0
TAG 56:8-20:4	0	0.217	0/0

TAG 56:8-22:6	0	0.356	0/0
TAG 58:10-20:4	0	0.307	0/0
TAG 58:10-22:6	0	0.040	0/0
TAG 58:9-20:4	0	0.511	0/0
TAG 58:9-22:6	0	0.435	0/0
FC	0	0.738	0/0
**LPC 14:0**	**500**	**2.237**	**10/0**
LPC 16:0	0	2.612	10/0
LPC 16:1	0	2.403	0/6
LPC 18:0	0	2.556	10/0
LPC 18:1	0	2.504	10/0
LPC 18:2	0	2.484	10/0
**LPC 20:4**	**441**	**2.545**	**0/10**
LPC 22:6	0	2.221	9/1
LPE 16:0	0	1.957	1/9
LPE 18:0	0	2.029	3/0
**LPE 18:1**	**16**	**2.166**	**0/9**
**LPE 20:4**	**500**	**2.512**	**10/0**
LPE 22:6	0	1.981	0/0
PC 32:0	0	0.299	0/0
PC 34:1	0	0.293	0/0
PC 34:2	0	0.058	0/0
PC 36:1	0	0.653	0/0
PC 36:2	0	0.342	0/0
PC 36:3	0	1.073	0/0
PC 36:4	0	0.484	0/0
PC 38:4	0	0.617	0/0
PC 38:5	0	1.048	0/0
PC 38:6	0	0.412	0/0
PC 40:6	0	0.413	0/0

PC 40:7	0	0.881	0/0
PE 34:1	0	0.578	0/0
PE 34:2	389	0.648	0/1
PE 36:1	0	0.260	0/0
PE 36:2	0	0.302	0/0
PE 36:3	0	0.321	0/0
PE 36:4	0	0.239	0/0
PE 38:4	0	0.033	0/0
PE 38:5	0	0.001	0/0
PE 38:6	0	0.433	0/0
PE 40:6	0	0.313	0/0

Results of the Lasso regression are presented as the number of times the respective lipid was selected after 500 runs, and those of the OPLS-DA analysis as VIP scores. Results of the random forest (Boruta) analysis are presented as the number of confirmed or tentative selections within 10 runs. Cer, ceramide; DAG, diacylglycerol; FC, free cholesterol; LPC, lysophosphatidylcholine; LPE, lysophosphatidylethanolamine; MG, monoacylglycerol; OPLS-DA, orthogonal partial least squares discriminant analysis; PC, phosphatidylcholine; PE, phosphatidylethanolamine; SM, sphingomyelin; TAG, triacylglycerol. VIP, variable of importance projection.

Supplemental Table S2. Differences in Lipid Serum Levels Between Patients Who Were Alive and Those Who Died in the Hospital Due to AHF.

	**Discovery cohort**				
**Lipids (pmol/mL)**	**Alive (N=281)**	**Dead (N=34)**	**Total (N=315)**	**P value (raw)**	**P value (BH adjusted)**
SM 16:0	15020 (12889-17860)	14184 (12470-16911)	14948 (12803-17801)	0.268	0.591
SM 16:1	3008 (2419-3727)	2533 (2143-3601)	2988 (2405-3727)	0.119	0.445
SM 18:0	2600 (2130-3126)	2212 (2059-2841)	2568 (2100-3101)	**0.021**	0.114
SM 20:0	578 (409-785)	458 (385-578)	563 (407-767)	**0.001**	**0.012**
SM 22:0	2587 (2036-3368)	2192 (1793-2776)	2530 (2012-3307)	**0.021**	0.114
SM 23:0	1199 (926-1527)	1148 (834-1300)	1179 (914-1517)	0.081	0.343
SM 24:0	1593 (1217-2078)	1388 (1068-1862)	1579 (1207-2030)	0.115	0.445
SM 24:1	6322 (5110-7969)	5727 (4972-6845)	6281 (5101-7877)	0.206	0.546
Cer d18:1/16:0	50 (40-62)	59 (39-71)	50 (40-63)	0.311	0.668
Cer d18:1/18:0	41 (29-55)	42 (30-60)	41 (29-56)	0.751	0.877
Cer d18:1/20:0	33 (23-48)	31 (24-49)	33 (23-48)	0.877	0.905
Cer d18:1/22:0	192 (129-284)	164 (128-271)	191 (128-283)	0.359	0.683
Cer d18:1/23:0	205 (134-287)	162 (121-242)	193 (134-286)	0.153	0.480
Cer d18:1/24:0	504 (327-727)	377 (259-543)	485 (310-721)	**0.010**	0.061
Cer d18:1/24:1	379 (288-529)	359 (255-545)	379 (280-531)	0.609	0.792
MG 16:0	773 (86-1439)	522 (38-1362)	749 (78-1422)	0.377	0.703
MG 18:0	502 (70-1170)	212 (35-1235)	467 (64-1175)	0.398	0.717
MG 18:1	532 (212-2790)	636 (169-1427)	537 (209-2688)	0.597	0.792
DAG 32:1-16:0	14 (6-37)	11 (4-27)	14 (6-36)	0.188	0.546
DAG 34:1-18:1	305 (208-475)	285 (206-402)	304 (206-475)	0.453	0.732
DAG 34:2-18:2	246 (162-388)	257 (139-408)	246 (160-389)	0.615	0.792
DAG 36:1-18:1	54 (37-85)	48 (29-72)	54 (35-82)	0.239	0.567
DAG 36:2-18:2	1343 (976-1922)	1197 (858-1815)	1340 (968-1920)	0.325	0.671
DAG 36:3-18:2	900 (604-1370)	863 (635-1167)	889 (606-1359)	0.359	0.683
DAG 36:4-18:2	128 (72-222)	119 (65-197)	128 (72-219)	0.561	0.792
TAG 48:0-16:0	2479 (479-7290)	2445 (20-5389)	2479 (449-7041)	0.135	0.451

TAG 48:1-16:0	4181 (1344-10498)	3753 (1214-7383)	4181 (1324-10045)	0.517	0.792
TAG 48:2-16:0	1760 (584-4537)	1569 (522-3222)	1707 (571-4384)	0.407	0.717
TAG 50:0-16:0	911 (170-2444)	999 (16-1506)	917 (149-2324)	0.091	0.368
TAG 50:1-16:0	29054 (9107-65518)	27997 (8601-47490)	28857 (8870-64476)	0.620	0.792
TAG 50:2-16:0	30734 (9143-61764)	25237 (8835-55793)	30424 (9141-61649)	0.720	0.852
TAG 50:3-16:0	6866 (2418-14718)	4599 (2517-12134)	6693 (2418-14647)	0.610	0.792
TAG 50:4-18:2	1502 (529-3772)	1225 (609-3102)	1485 (530-3753)	0.439	0.724
TAG 52:0-18:0	209 (20-705)	209 (15-425)	209 (19-684)	0.133	0.451
TAG 52:1-18:1	3531 (1122-7691)	3176 (1242-5761)	3443 (1136-7459)	0.772	0.878
TAG 52:2-18:1	119361 (30732-240191)	95785 (34792-210495)	117738 (31370-232691)	0.639	0.805
TAG 52:3-18:1	87256 (24248-174506)	62775 (25867-137407)	83810 (24362-173126)	0.702	0.850
TAG 52:4-18:2	45811 (15563-105133)	48218 (16887-77573)	46099 (15709-103220)	0.787	0.878
TAG 52:5-18:2	3352 (1344-7547)	2499 (1282-6677)	3154 (1331-7435)	0.400	0.717
TAG 54:1-18:1	274 (77-752)	257 (87-713)	274 (77-747)	0.850	0.894
TAG 54:2-18:1	5622 (2189-12500)	7454 (2834-11441)	5807 (2222-12426)	0.593	0.792
TAG 54:3-18:1	54437 (14714-105737)	42572 (17879-99656)	54000 (15263-105350)	0.955	0.975
TAG 54:3-18:2	6236 (2045-12719)	6825 (2236-12602)	6302 (2059-12719)	0.578	0.792
TAG 54:4-18:2	20293 (6046-38570)	16969 (7194-33729)	19770 (6081-38418)	0.826	0.880
TAG 54:5-18:2	17365 (6749-38060)	17723 (6662-28761)	17455 (6720-37752)	0.707	0.850
TAG 54:5-20:4	5592 (1835-11848)	3763 (2227-11270)	5223 (1840-11902)	0.523	0.792

TAG 54:6-18:2	5374 (2283-13030)	5660 (1881-11153)	5376 (2261-12989)	0.535	0.792
TAG 54:6-20:4	4643 (1858-9826)	3721 (1535-7051)	4273 (1687-9754)	0.609	0.792
TAG 54:7-18:2	254 (145-649)	243 (103-411)	252 (141-645)	0.239	0.567
TAG 56:2-18:1	89 (29-191)	105 (26-171)	94 (29-187)	0.978	0.988
TAG 56:3-18:1	527 (169-1087)	521 (207-993)	527 (174-1087)	0.818	0.880
TAG 56:4-18:2	309 (97-651)	262 (135-558)	309 (100-648)	0.990	0.990
TAG 56:5-18:2	668 (195-1305)	568 (260-1183)	634 (199-1302)	0.857	0.894
TAG 56:6-20:4	4445 (1327-8809)	2840 (1763-6972)	4318 (1354-8757)	0.509	0.792
TAG 56:7-20:4	3886 (1505-8221)	2671 (1411-5412)	3706 (1502-7940)	0.250	0.578
TAG 56:8-20:4	1008 (491-2187)	834 (417-1517)	974 (481-2157)	0.239	0.567
TAG 56:8-22:6	1407 (519-2940)	1513 (566-3711)	1413 (523-2989)	0.694	0.850
TAG 58:10-20:4	160 (63-351)	113 (54-214)	148 (62-343)	0.208	0.546
TAG 58:10-22:6	57 (11-207)	82 (12-209)	59 (11-207)	0.820	0.880
TAG 58:9-20:4	240 (100-544)	151 (78-361)	223 (98-539)	0.131	0.451
TAG 58:9-22:6	418 (180-1060)	542 (247-1045)	435 (182-1058)	0.559	0.792
FC	169319 (138426-209500)	162699 (141339-193423)	169199 (138542-208899)	0.787	0.878
LPC 14:0	67 (43-95)	43 (27-58)	62 (41-93)	**<0.001**	**<0.001**
LPC 16:0	18327 (14483-22736)	13638 (9568-17582)	17946 (13844-21937)	**<0.001**	**<0.001**
LPC 16:1	302 (213-395)	220 (159-309)	294 (206-391)	**<0.001**	**<0.001**
LPC 18:0	11331 (8649-13956)	7795 (5444-11476)	11093 (8433-13500)	**<0.001**	**0.001**
LPC 18:1	5199 (4113-6630)	3399 (2937-4761)	5039 (3901-6552)	**<0.001**	**<0.001**
LPC 18:2	5049 (3482-6808)	2692 (1766-3826)	4762 (3244-6668)	**<0.001**	**<0.001**
LPC 20:4	1763 (1198-2274)	1051 (690-1465)	1656 (1089-2234)	**<0.001**	**<0.001**
LPC 22:6	313 (212-430)	217 (150-304)	305 (206-417)	**<0.001**	**<0.001**
LPE 16:0	221 (168-295)	172 (137-219)	217 (164-291)	**0.002**	**0.013**
LPE 18:0	263 (205-348)	203 (163-301)	255 (195-340)	**0.004**	**0.029**
LPE 18:1	202 (138-286)	125 (108-178)	194 (132-281)	**<0.001**	**<0.001**
LPE 20:4	227 (167-313)	138 (92-191)	219 (155-305)	**<0.001**	**<0.001**
LPE 22:6	142 (91-191)	92 (69-134)	136 (88-185)	**<0.001**	**0.003**

PC 32:0	2803 (2286-3454)	3148 (2501-3636)	2858 (2302-3482)	0.183	0.546
PC 34:1	30021 (22913-36446)	27973 (22672-34577)	29709 (22871-36188)	0.340	0.674
PC 34:2	43201 (30639-51952)	42249 (34619-48709)	42967 (32190-51588)	0.422	0.718
PC 36:1	3466 (2644-4446)	3123 (2510-4083)	3431 (2632-4335)	0.203	0.546
PC 36:2	27675 (20823-33820)	26329 (20236-29145)	27249 (20685-32949)	0.151	0.480
PC 36:3	19015 (14939-24598)	16939 (13093-21551)	18712 (14645-24174)	**0.009**	0.060
PC 36:4	15416 (11555-20578)	14589 (10195-17132)	15269 (11446-20199)	**0.036**	0.173
PC 38:4	12514 (9429-16343)	11698 (8923-13994)	12471 (9263-15996)	0.061	0.283
PC 38:5	5764 (4707-7290)	5407 (4703-6028)	5743 (4707-7098)	**0.033**	0.171
PC 38:6	8233 (6458-10825)	8659 (6569-9592)	8384 (6462-10541)	0.810	0.880
PC 40:6	2076 (1574-2882)	2123 (1502-2679)	2080 (1555-2864)	0.776	0.878
PC 40:7	374 (280-455)	337 (284-408)	370 (280-452)	0.207	0.546
PE 34:1	321 (223-466)	335 (230-586)	324 (223-469)	0.235	0.567
PE 34:2	777 (508-1102)	932 (615-1404)	799 (514-1137)	0.065	0.287
PE 36:1	309 (224-443)	328 (224-524)	314 (224-460)	0.602	0.792
PE 36:2	1815 (1272-2603)	2146 (1435-2975)	1832 (1279-2638)	0.260	0.586
PE 36:3	553 (378-773)	631 (397-866)	560 (378-789)	0.440	0.724
PE 36:4	852 (582-1265)	866 (605-1402)	852 (588-1282)	0.414	0.717
PE 38:4	3113 (2216-4563)	3218 (2413-4752)	3113 (2223-4620)	0.710	0.850
PE 38:5	591 (417-836)	582 (459-879)	591 (417-852)	0.595	0.792
PE 38:6	874 (539-1308)	878 (678-1466)	874 (553-1324)	0.317	0.668
PE 40:6	606 (385-939)	640 (458-1144)	612 (398-949)	0.338	0.674

	**Validation cohort**				
**Lipids (pmol/mL)**	**Alive (N=118)**	**Dead (N=21)**	**Total (N=139)**	**P value (raw)**	**P value (BH adjusted)**
SM 16:0	8115 (7080-8887)	8040 (7041-8543)	8072 (7061-8882)	0.842	0.925

SM 16:1	1117 (961-1314)	1026 (887-1313)	1103 (953-1315)	0.246	0.398
SM 18:0	916 (847-1077)	951 (790-1102)	922 (838-1084)	0.926	0.979
SM 20:0	532 (455-625)	493 (380-544)	521 (453-606)	**0.007**	**0.030**
SM 22:0	1625 (1320-1951)	1287 (1134-1541)	1596 (1300-1914)	**<0.001**	**0.005**
SM 23:0	732 (644-887)	556 (462-623)	723 (593-865)	**<0.001**	**0.001**
SM 24:0	1102 (876-1340)	802 (734-940)	1059 (824-1302)	**<0.001**	**0.007**
SM 24:1	3641 (3223-4071)	3642 (3296-4224)	3642 (3226-4104)	0.566	0.743
Cer d18:1/16:0	29 (25-36)	29 (24-38)	29 (24-37)	0.636	0.801
Cer d18:1/18:0	16 (12-20)	17 (10-23)	16 (12-21)	0.716	0.857
Cer d18:1/20:0	14 (11-18)	13 (9-20)	14 (11-18)	0.821	0.915
Cer d18:1/22:0	91 (70-126)	84 (55-113)	89 (67-126)	0.145	0.261
Cer d18:1/23:0	88 (63-121)	61 (52-76)	82 (60-117)	**0.004**	**0.018**
Cer d18:1/24:0	277 (199-399)	186 (125-259)	265 (190-375)	**0.003**	**0.013**
Cer d18:1/24:1	149 (116-183)	143 (108-197)	149 (114-184)	0.988	0.995
MG 16:0	1463 (1197-1912)	1352 (1172-1466)	1447 (1192-1809)	0.186	0.323
MG 18:0	640 (530-781)	681 (515-718)	641 (527-777)	0.848	0.925
MG 18:1	498 (322-1712)	465 (292-1036)	493 (306-1676)	0.412	0.603
DAG 32:1-16:0	14 (8-21)	8 (7-12)	12 (7-20)	**0.010**	**0.035**
DAG 34:1-18:1	145 (109-206)	113 (77-154)	140 (104-202)	0.070	0.165
DAG 34:2-18:2	101 (70-134)	70 (59-84)	93 (68-131)	**0.012**	**0.038**
DAG 36:1-18:1	24 (17-37)	22 (15-31)	24 (17-37)	0.416	0.603
DAG 36:2-18:2	470 (339-638)	421 (255-514)	462 (326-625)	0.207	0.352
DAG 36:3-18:2	244 (159-332)	171 (151-243)	230 (154-330)	**0.041**	0.104
DAG 36:4-18:2	102 (67-178)	70 (46-108)	98 (63-161)	**0.023**	0.062
TAG 48:0-16:0	217 (130-367)	136 (114-309)	209 (126-358)	0.094	0.196
TAG 48:1-16:0	270 (164-456)	196 (129-291)	263 (162-417)	**0.038**	0.099
TAG 48:2-16:0	114 (68-182)	65 (46-105)	100 (63-175)	**0.005**	**0.024**
TAG 50:0-16:0	126 (68-267)	77 (60-191)	124 (65-253)	0.103	0.196
TAG 50:1-16:0	1968 (1383-2684)	1622 (1177-2355)	1878 (1295-2598)	0.363	0.551
TAG 50:2-16:0	1576 (1229-2311)	1326 (1007-1631)	1547 (1213-2218)	0.086	0.190
TAG 50:3-16:0	364 (254-503)	251 (183-321)	338 (247-487)	**0.011**	**0.035**
TAG 50:4-18:2	87 (53-138)	53 (40-77)	80 (50-135)	**0.002**	**0.013**
TAG 52:0-18:0	47 (24-98)	31 (19-63)	44 (22-95)	0.101	0.196

TAG 52:1-18:1	365 (238-631)	326 (215-601)	363 (238-629)	0.423	0.603
TAG 52:2-18:1	6396 (4916-9171)	6795 (4553-8751)	6410 (4791-9157)	0.600	0.765
TAG 52:3-18:1	3714 (3032-5261)	3391 (2702-4277)	3659 (2996-5054)	0.101	0.196
TAG 52:4-18:2	2201 (1538-3169)	1571 (1169-2234)	2105 (1490-2969)	**0.022**	0.061
TAG 52:5-18:2	172 (105-272)	102 (76-150)	156 (101-258)	**0.001**	**0.009**
TAG 54:1-18:1	44 (26-74)	39 (23-78)	40 (25-74)	0.414	0.603
TAG 54:2-18:1	805 (579-1076)	804 (467-1138)	804 (573-1114)	0.962	0.990
TAG 54:3-18:1	2770 (2075-3492)	2727 (2278-3881)	2763 (2079-3560)	0.690	0.837
TAG 54:3-18:2	378 (276-485)	373 (253-525)	376 (275-490)	0.897	0.967
TAG 54:4-18:2	833 (608-1053)	748 (622-1029)	824 (609-1047)	0.567	0.743
TAG 54:5-18:2	648 (485-929)	547 (360-893)	638 (448-918)	0.101	0.196
TAG 54:5-20:4	177 (135-243)	159 (129-213)	174 (134-232)	0.229	0.377
TAG 54:6-18:2	224 (148-341)	145 (91-248)	214 (131-315)	**0.012**	**0.038**
TAG 54:6-20:4	165 (114-226)	122 (82-201)	160 (110-221)	0.056	0.138
TAG 54:7-18:2	18 (11-29)	8 (5-14)	17 (9-27)	**<0.001**	**0.001**
TAG 56:2-18:1	18 (12-26)	18 (11-25)	18 (11-26)	0.584	0.755
TAG 56:3-18:1	64 (46-83)	73 (52-102)	65 (46-87)	0.308	0.474
TAG 56:4-18:2	21 (17-26)	19 (16-34)	21 (17-27)	0.795	0.908
TAG 56:5-18:2	20 (15-28)	18 (14-33)	20 (15-28)	0.760	0.878
TAG 56:6-20:4	120 (88-161)	111 (89-142)	120 (88-159)	0.437	0.606
TAG 56:7-20:4	100 (67-136)	76 (49-121)	97 (63-135)	0.180	0.317
TAG 56:8-20:4	40 (26-63)	25 (14-45)	38 (23-61)	0.092	0.196
TAG 56:8-22:6	59 (32-92)	52 (34-64)	59 (33-86)	0.132	0.241
TAG 58:10-20:4	9 (4-14)	5 (3-13)	9 (4-14)	0.099	0.196
TAG 58:10-22:6	9 (5-13)	7 (5-9)	9 (5-13)	0.084	0.189
TAG 58:9-20:4	10 (6-17)	7 (4-13)	9 (6-16)	0.131	0.241
TAG 58:9-22:6	20 (10-31)	18 (12-24)	19 (11-29)	0.489	0.669
FC	112653 (93633-140328)	104631 (84274-118003)	109392 (93169-132871)	0.082	0.189
LPC 14:0	50 (34-72)	28 (19-42)	47 (31-70)	**<0.001**	**0.002**
LPC 16:0	6855 (5656-8159)	4199 (3697-5787)	6645 (5158-8091)	**<0.001**	**0.001**
LPC 16:1	153 (114-212)	84 (68-121)	143 (103-201)	**<0.001**	**0.006**
LPC 18:0	2517 (1931-3162)	1715 (1235-2204)	2366 (1796-3080)	**<0.001**	**0.005**

LPC 18:1	1369 (1060-1728)	962 (783-1112)	1316 (976-1710)	**<0.001**	**0.006**
LPC 18:2	1615 (1292-2203)	924 (683-1188)	1488 (1056-2101)	**<0.001**	**<0.001**
LPC 20:4	477 (343-711)	286 (231-488)	459 (325-686)	**0.001**	**0.008**
LPC 22:6	80 (61-110)	52 (39-72)	75 (57-107)	**<0.001**	**0.001**
LPE 16:0	97 (76-123)	82 (59-113)	94 (73-123)	0.211	0.354
LPE 18:0	111 (84-134)	92 (74-143)	110 (83-135)	0.273	0.426
LPE 18:1	64 (47-89)	43 (35-59)	61 (45-86)	**0.001**	**0.008**
LPE 20:4	76 (58-108)	53 (41-82)	72 (53-100)	**<0.001**	**0.008**
LPE 22:6	49 (35-65)	43 (28-45)	46 (34-63)	**0.007**	**0.030**
PC 32:0	1344 (1140-1539)	1607 (1295-1660)	1385 (1147-1572)	0.060	0.144
PC 34:1	16120 (14014-19192)	16152 (14585-20139)	16135 (14054-19314)	0.930	0.979
PC 34:2	28194 (23511-33264)	24923 (21247-28277)	27307 (23075-32913)	**0.009**	**0.035**
PC 36:1	2517 (2157-3164)	2496 (1864-2815)	2498 (2142-3060)	0.537	0.723
PC 36:2	15974 (13126-19334)	13836 (12092-15690)	15528 (12827-18986)	**0.010**	**0.035**
PC 36:3	7958 (6363-9563)	6169 (5458-7309)	7610 (6133-9389)	**0.002**	**0.009**
PC 36:4	10262 (7912-12896)	8023 (5973-9882)	9766 (7517-12613)	**0.016**	0.047
PC 38:4	7400 (5991-9676)	5327 (4579-7684)	7187 (5761-9357)	**0.014**	**0.041**
PC 38:5	2382 (1778-2788)	1807 (1287-2064)	2250 (1764-2739)	**0.002**	**0.012**
PC 38:6	2491 (1822-3146)	1724 (1444-2172)	2298 (1719-3046)	**0.005**	**0.024**
PC 40:6	974 (773-1295)	718 (583-915)	950 (680-1283)	**0.020**	0.057
PC 40:7	139 (105-182)	106 (86-133)	133 (95-178)	**0.010**	**0.035**
PE 34:1	93 (65-125)	101 (80-131)	96 (67-126)	0.264	0.421
PE 34:2	161 (125-224)	210 (124-235)	166 (124-228)	0.429	0.604
PE 36:1	67 (47-92)	64 (43-101)	65 (47-96)	0.969	0.990
PE 36:2	335 (259-453)	381 (241-466)	338 (247-461)	0.753	0.878
PE 36:3	96 (70-128)	106 (76-126)	97 (70-128)	0.805	0.908
PE 36:4	149 (103-196)	145 (120-218)	147 (108-200)	0.677	0.837
PE 38:4	512 (352-699)	447 (371-611)	512 (360-695)	0.687	0.837
PE 38:5	120 (81-170)	109 (92-166)	119 (82-170)	0.939	0.979

PE 38:6	176 (112-274)	183 (120-248)	180 (119-264)	0.750	0.878
PE 40:6	122 (73-191)	132 (70-176)	122 (72-188)	0.995	0.995

Data are presented as median and interquartile range (Q1-Q3). Differences between AHF patients who were alive and those who died in hospital were tested with the Brunner-Munzel test. P-values < 0.05 are considered significant and are depicted in bold.

Different responses of internal standards and different software used for the processing of the MS data preclude a direct comparison of the absolute concentrations of lipids between discovery and validation cohort.

BH, Benjamini-Hochberg; Cer, ceramide; DAG, diacylglycerol; FC, free cholesterol; LPC, lysophosphatidylcholine; LPE, lysophosphatidylethanolamine; MG, monoacylglycerol; PC, phosphatidylcholine; PE, phosphatidylethanolamine; SM, sphingomyelin; TAG, triacylglycerol.

Supplemental Table S3. Univariable Logistic Regression Analyses of Clinical and Laboratory Parameters as Predictors of In-Hospital Mortality in the Discovery Cohort of AHF Patients.

	**OR (95% CI)**	**p-value**	**Event/N**
ADHERE (low)	0.29 (0.14-0.60)	**0.001**	34/315
GWTG-HF	1.10 (1.05-1.16)	**<0.001**	33/314
OPTIMIZE-HF	1.11 (1.05-1.17)	**<0.001**	27/303
Age (years)	1.03 (0.99-1.06)	0.176	34/315
Sex (female)	1.04 (0.50-2.13)	0.906	34/315
BMI (kg/m^2)	1.05 (1.00-1.11)	**0.042**	34/315
CRP (mg/mL)	1.01 (1.00-1.02)	**0.002**	34/315
HDL-C (mmol/L)	0.19 (0.06-0.57)	**0.005**	34/315
NT-proBNP (pg/mL)	1.00 (1.00-1.00)	**0.001**	34/315
ALT (U/L)	1.00 (1.00-1.01)	**<0.001**	34/315
BUN (mmol/L)	1.09 (1.03-1.16)	**0.002**	33/314
Chloride (mmol/L)	0.90 (0.84-0.95)	**<0.001**	34/315
Hemoglobin (g/L)	0.98 (0.96-0.99)	**0.008**	34/315
MAP (mm Hg)	0.97 (0.95-0.99)	**0.001**	34/315
eGFR (ml/min/1.73 m^2)	0.97 (0.95-0.99)	**0.004**	34/315
Enlarged liver	2.39 (1.11-5.59)	**0.032**	34/315

P-values < 0.05 are considered significant and are depicted in bold. ADHERE, Acute Decompensated Heart Failure National Registry; ALT, alanine aminotransferase; BMI, body mass index; BUN, blood urea nitrogen; CRP, C-reactive protein; eGFR, estimated glomerular filtration rate; GWTG-HF, Get With The Guidelines Heart Failure; HDL-C, high-density lipoprotein cholesterol; MAP, mean arterial pressure; N, number of observations; NT-proBNP, N-terminal pro brain natriuretic peptide; OPTIMIZE-HF, Organized program to Initiate Lifesaving Treatment in Hospitalized Patients With Heart Failure; OR, odds ratio.

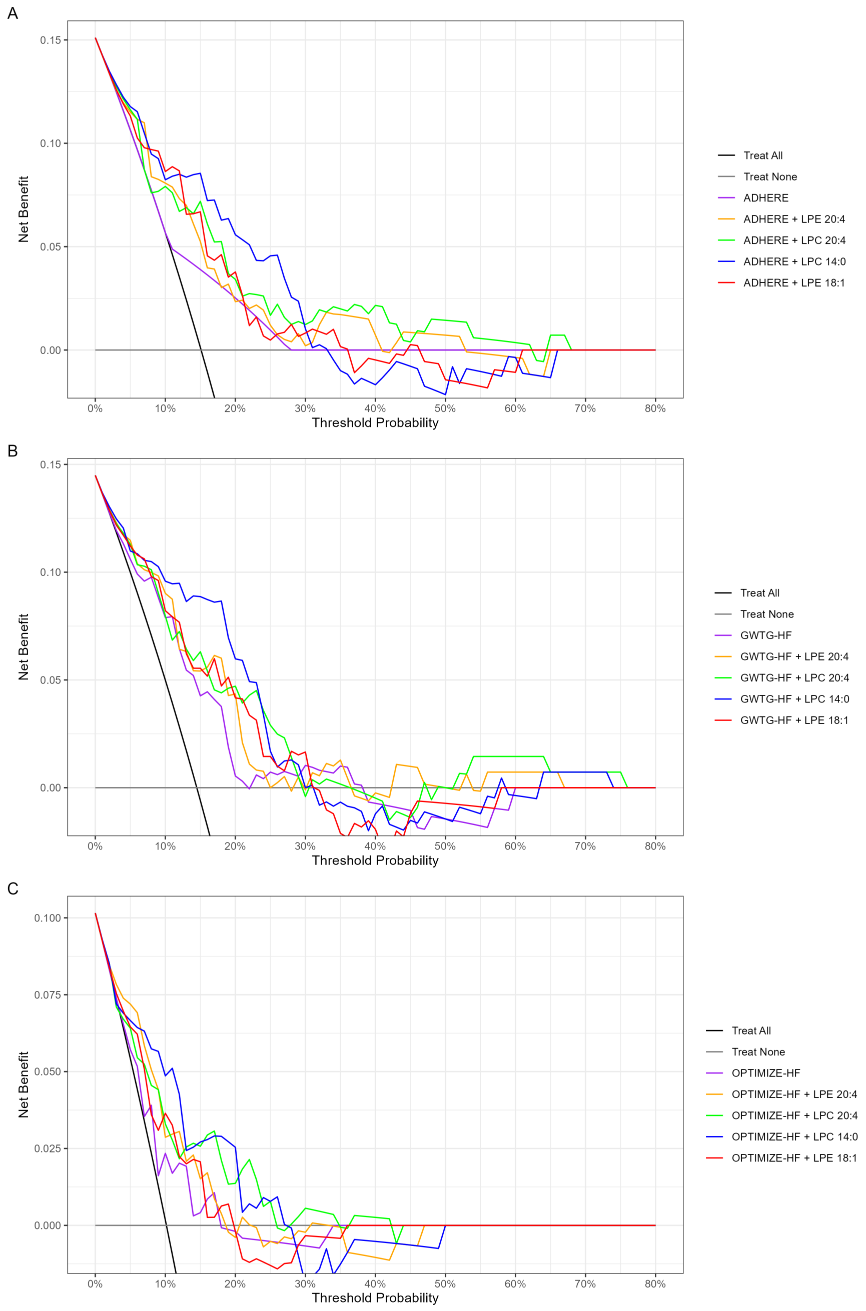

Supplemental Figure S1. Decision curve analysis comparing net benefit of baseline risk scores with lipid-enhanced risk models for predicting in-hospital mortality of patients with AHF in the validation cohort. ADHERE, Acute Decompensated Heart Failure National Registry; AHF, acute heart failure; GWTG-HF, Get With The Guidelines Heart Failure; LPC, lysophosphatidylcholine; LPE, lysophosphatidylethanolamine; OPTIMIZE-HF, Organized Program to Initiate Lifesaving Treatment in Hospitalized Patients With Heart Failure.

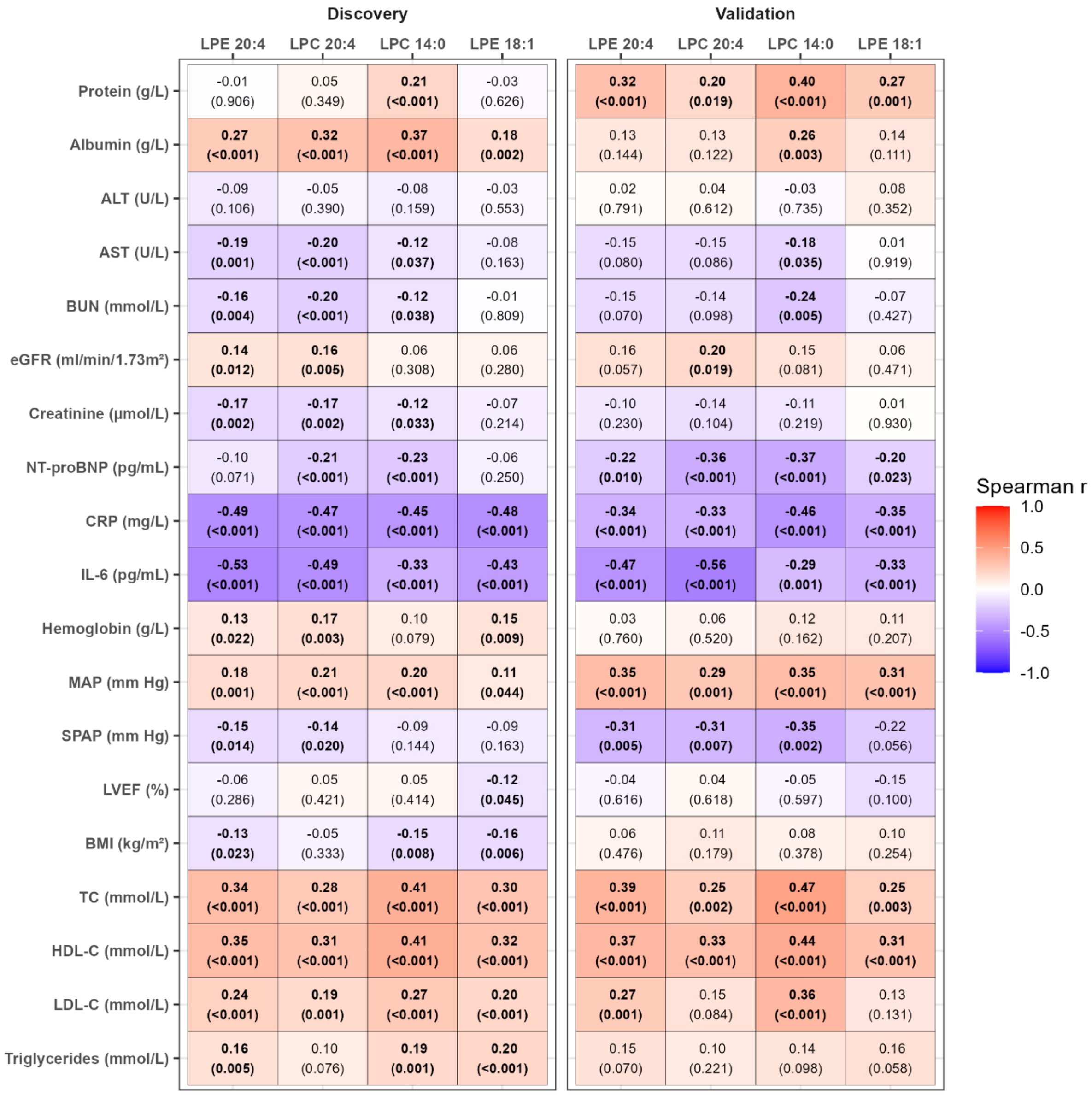

Supplemental Figure S2. Heatmap of correlations of the lipids' serum levels with laboratory and clinical parameters. Values presented are the Spearman correlation coefficients. P-values < 0.05 were considered significant and are depicted in bold. ALT, alanine aminotransferase; AST, aspartate aminotransferase; BMI, body mass index; BUN, blood urea nitrogen; CK, creatine kinase; CRP, C-reactive protein; eGFR, estimated glomerular filtration rate; HDL-C, high-density lipoprotein cholesterol; hsTnI, high-sensitivity troponin I; IL-6, interleukin 6; LDH, lactate dehydrogenase; LDL-C, low-density lipoprotein cholesterol; LVEF, left ventricular ejection fraction; MAP, mean arterial pressure; NT-proBNP, N-terminal pro brain natriuretic peptide; SPAP, systolic pulmonary artery pressure; TC, total cholesterol.
