# Supplementary material for: Low Serum Lysophospholipids Predict Increased In‐Hospital Mortality in Patients With Acute Heart Failure
Source: J Am Heart Assoc. 2026 Jan 19;15(2):e043828. doi: 10.1161/JAHA.125.043828 (PMC12919526; doi:10.1161/JAHA.125.043828)
Supplement: Supplementary file 2 — Tables S1–S3 Figures S1–S2 [file JAH3-15-e043828-s002.pdf]

STROBE Statement—checklist of items that should be included in reports of observational studies

	Item No.	Recommendation	Page No.	Relevant text from manuscript
**Title and abstract**	1	(*a*) Indicate the study's design with a commonly used term in the title or the abstract	3	patients hospitalized due to AHF that were prospectively enrolled in two observational studies
		(*b*) Provide in the abstract an informative and balanced summary of what was done and what was found	3	Please see the abstract
**Introduction**				
Background/rationale	2	Explain the scientific background and rationale for the investigation being reported	6	…no study has examined the association of serum lipids with the outcome of hospitalized AHF patients.
Objectives	3	State specific objectives, including any prespecified hypotheses	7	Therefore, we employed mass spectrometry (MS)-based serum lipidomics to identify lipids with predictive ability for in-hospital mortality in AHF patients.
**Methods**				
Study design	4	Present key elements of study design early in the paper	7	Patients with signs and symptoms of AHF requiring hospitalisation, from both discovery and validation cohort, were enrolled prospectively and consecutively in observational studies, as they presented to the Emergency Department

Setting	5	Describe the setting, locations, and relevant dates, including periods of recruitment, exposure, follow-up, and data collection	7	… as they presented to the Emergency Department of the Sisters of Charity University Hospital Centre in Zagreb, Croatia. Patients in the discovery cohort (N=315) were recruited between March 2018 and February 2021 (14) and in the validation cohort (N=152) between November 2013 and February 2015 (15). Collection of patient history, physical examination, and venous blood collection were performed at the time the patients presented to the emergency department, prior to the application of any medication.
Participants	6	(*a*) *Cohort study*—Give the eligibility criteria, and the sources and methods of selection of participants. Describe methods of follow-up<br>*Case-control study*—Give the eligibility criteria, and the sources and methods of case ascertainment and control selection. Give the rationale for the choice of cases and controls<br>*Cross-sectional study*—Give the eligibility criteria, and the sources and methods of selection of participants	7	The study protocols are described in detail in the previous reports (14,15). From the validation cohort, 139 blood samples were available for the present lipidomics analyses. In-hospital mortality was the primary outcome.
		(*b*) *Cohort study*—For matched studies, give matching criteria and number of exposed and unexposed<br>*Case-control study*—For matched studies, give matching criteria and the number of controls per case	N/A	

Variables	7	Clearly define all outcomes, exposures, predictors, potential confounders, and effect modifiers. Give diagnostic criteria, if applicable	10	See Statistical analysis
Data sources/ measurement	8*	For each variable of interest, give sources of data and details of methods of assessment (measurement). Describe comparability of assessment methods if there is more than one group	7 Refs 14, 15	
Bias	9	Describe any efforts to address potential sources of bias	9	Finally, we consolidated the results from the three selection methods using the R-package TopKSignal (20) to obtain one final ranking of lipids. TopKSignal computes the consensus rank using a signal-plus-noise model for signal reconstruction, which aims to recover the true underlying ranking from a set of noisy rankings. In contrast, standard rank aggregation methods typically do not account for the specific noise characteristics in the ranking data, making them more susceptible to errors or inconsistencies. Additionally, the approach provides measures of (un)certainty for the estimated consensus signals, which are valuable for assessing the reliability of results from feature selection processes. We have created ten replicates of the OPLS-DA and LASSO derived feature ranks while resolving ties randomly. The resulting 30 feature rankings (10 per method, including the 10 original Boruta rankings) were then used as input for TopKSignal.
Study size	10	Explain how the study size was arrived at	Refs 14,15	

Continued on next page

Quantitative variables	11	Explain how quantitative variables were handled in the analyses. If applicable, describe which groupings were chosen and why	10	Data is descriptively summarized as median and interquartile range (Q1, Q3)… Group differences were assessed with the Mann-Whitney U test…
Statistical methods	12	(*a*) Describe all statistical methods, including those used to control for confounding	10	
		(*b*) Describe any methods used to examine subgroups and interactions	N/A	
		(*c*) Explain how missing data were addressed	8,9	Lipid data was log2-transformed and values under the detection limit were imputed using the QRILC algorithm, a missing data imputation method that performs the imputation of left-censored missing data using random draws from a truncated distribution with parameters estimated using quantile regression, that is implemented in the R package imputeLCMD.
		(*d*) *Cohort study*—If applicable, explain how loss to follow-up was addressed<br>*Case-control study*—If applicable, explain how matching of cases and controls was addressed<br>*Cross-sectional study*—If applicable, describe analytical methods taking account of sampling strategy	N/A	
		(*e*) Describe any sensitivity analyses	N/A	
**Results**				
Participants	13*	(a) Report numbers of individuals at each stage of study—eg numbers potentially eligible, examined for eligibility, confirmed eligible, included in the study, completing follow-up, and analysed	Table 1, Refs 14,15	
		(b) Give reasons for non-participation at each stage	N/A	
		(c) Consider use of a flow diagram	Refs 14,15	
Descriptive data	14*	(a) Give characteristics of study participants (eg demographic, clinical, social) and information on exposures and potential confounders	10,11, Table 1 and Refs 14,15	
		(b) Indicate number of participants with missing data for each variable of interest	N/A	
		(c) *Cohort study*—Summarise follow-up time (eg, average and total amount)	Table 1	
Outcome data	15*	*Cohort study*—Report numbers of outcome events or summary measures over time	Table 1	

		Case-control study—Report numbers in each exposure category, or summary measures of exposure	*N/A*
		Cross-sectional study—Report numbers of outcome events or summary measures	*N/A*
Main results	16	(*a*) Give unadjusted estimates and, if applicable, confounder-adjusted estimates and their precision (eg, 95% confidence interval). Make clear which confounders were adjusted for and why they were included	11, Fig.3
		(*b*) Report category boundaries when continuous variables were categorized	N/A
		(*c*) If relevant, consider translating estimates of relative risk into absolute risk for a meaningful time period	N/A

Continued on next page

Other analyses	17	Report other analyses done—eg analyses of subgroups and interactions, and sensitivity analyses	12	ROC curve analyses, cNRI
**Discussion**				
Key results	18	Summarise key results with reference to study objectives	12	In the present study, we show for the first time that the four lipids LPE 20:4, LPC 20:4, LPC 14:0, and LPE 18:1, identified by serum lipidomics and comprehensive statistical approaches, exhibit predictive ability for in-hospital mortality in patients with AHF.
Limitations	19	Discuss limitations of the study, taking into account sources of potential bias or imprecision. Discuss both direction and magnitude of any potential bias	14,15	The study cohorts differed in some inherent features, such as the disease chronicity and severity, as well as the extent of congestion and inflammation. The cohorts also differed in the duration of sample storage between the sample collection and MS analysis (4 and 8 years from the beginning of the discovery and validation cohort, respectively). In addition, the study design does not allow for the investigation of causality and underlying mechanisms. Since the lipidomics data provide only a snapshot of lipid levels at admission, the dynamics of lipids could not be examined. Due to the fact that the patients' nutritional state at admission was unknown, its impact on the lipid levels could not

				be examined. Since all patients were enrolled in a single centre, the generalizability of the present data is limited. Despite its better sensitivity and selectivity, the limited lipid coverage of the targeted lipidomics approach might have precluded identification of additional potential prognostic lipids.
Interpretation	20	Give a cautious overall interpretation of results considering objectives, limitations, multiplicity of analyses, results from similar studies, and other relevant evidence	14,15	The identified tested lipids exhibited a moderate predictive ability. Consideration of the single lipids, as well as of the panel of these lipids, on top of the established risk scores improved the discrimination and classification for in-hospital mortality in patients with AHF. It is conceivable that a multifactorial regulation of their bioavailability and a broad spectrum of bioactivities underlie the ability of the identified lipids to capture and reflect the severity of the HF pathophysiology. Accordingly, the identified lipids are promising biomarkers whose prognostic performance and clinical utility must be further validated in larger cohorts of AHF patients.
Generalisability	21	Discuss the generalisability (external validity) of the study results	14	Since all patients were enrolled in a single centre, the generalizability of the present data is limited.
**Other information**				
Funding	22	Give the source of funding and the role of the funders for the present study and, if applicable, for the 15original study on which the present article is based		The work was supported by the Austrian Science Fund (FWF), the Austrian Research Promotion

	Agency (FFG), the Integrative Metabolism Research Center Graz, the Austrian Infrastructure Program 2016/2017, the Styrian Government, the City of Graz and BioTechMed-Graz. This project was also funded in part by the FFG and the European Union (EFRE). The funders had no role in study design, in the collection, analysis and interpretation of data, as well as in the writing of the report and in the decision to submit the article for publication. For open access purposes, the author has applied a CC BY public copyright license to any author accepted manuscript version arising from this submission.

*Give information separately for cases and controls in case-control studies and, if applicable, for exposed and unexposed groups in cohort and cross-sectional studies.

Note: An Explanation and Elaboration article discusses each checklist item and gives methodological background and published examples of transparent reporting. The STROBE checklist is best used in conjunction with this article (freely available on the Web sites of PLoS Medicine at http://www.plosmedicine.org/, Annals of Internal Medicine at http://www.annals.org/, and Epidemiology at http://www.epidem.com/). Information on the STROBE Initiative is available at www.strobe-statement.org.
